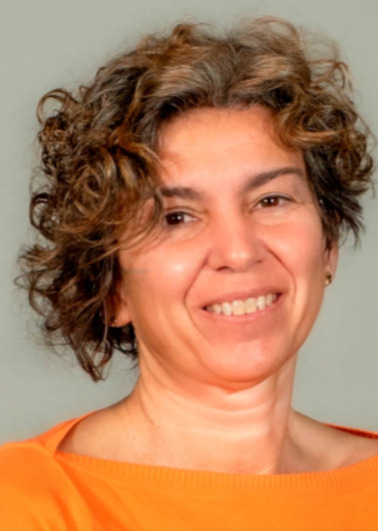# Fabrication Strategies Towards Hydrogels for Biomedical Application: Chemical and Mechanical Insights

**DOI:** 10.1002/asia.202200797

**Published:** 2022-10-05

**Authors:** Federico Acciaretti, Simone Vesentini, Laura Cipolla

**Affiliations:** ^1^ Department of Biotechnology and Biosciences University of Milano – Bicocca Piazza della Scienza 2 20126 Milano Italy; ^2^ Department of Electronics Information and Bioengineering Politecnico di Milano (Italy) Piazza Leonardo da Vinci 32 20133 Milano Italy

**Keywords:** hydrogels, cross-linking, dynamic reactions, smart hydrogels, stimuli responsiveness

## Abstract

This review aims at giving selected chemical and mechanical insights on design criteria that should be taken into account in hydrogel production for biomedical applications. Particular emphasis will be given to the chemical aspects involved in hydrogel design: macromer chemical composition, cross‐linking strategies and chemistry towards “conventional” and smart/stimuli responsive hydrogels. Mechanical properties of hydrogels in view of regenerative medicine applications will also be considered.

## Graphical abstract

**Insights in chemical** and mechanical criteria for hydrogel designs for biomedical applications are considered. Particular attention is given to the chemical aspects of macromers and cross‐linkers used for hydrogel fabrication.

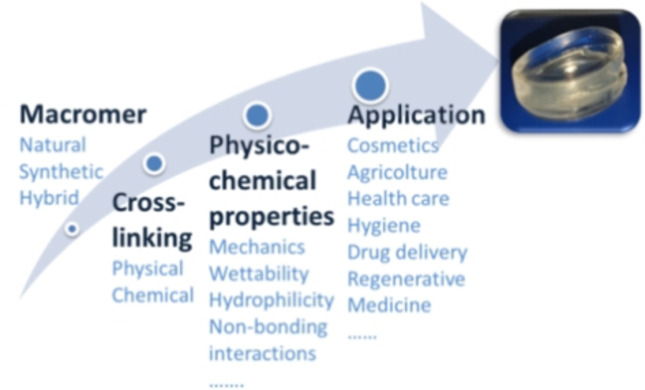

## 1 Introduction

Hydrogels can be defined as three‐dimensional polymeric networks able to swell and absorb high amounts of water without dissolving in short times.^[1]^ Cross links among the polymer macromers used to create the gel network are usually needed, to provide resistance to degradation in water and to impart mechanical features. Chemical structure, concentration, and cross‐linking strategies are key issues determining the final mechanical, physico‐chemical, and biological properties of the hydrogels and their application.

Hydrogels were first proposed as suitable “plastics” for biomedical applications by Wichterle and Lim in the late 50’s, as 3D structures able to absorb water (thus possessing hydrophilic properties), biologically inert (cross‐linking is desirable for absorption prevention), stable to degradation, and permeable to metabolites.^[2]^ In their seminal paper, they propose the use of polyhydroxyethyl methacrylate (pHEMA) hydrogels as filling after enucleation of the eye, contact lenses, and artificial arteries. While in their infancy, biological and chemical inertness were key requirements of hydrogels for biomedical applications. Upgrades of hydrogels especially for the biomedical sector came in the 80’s through the use of these materials as 3D scaffolds for cell culture^[3]^ and, later on, with the design of bioactive hydrogels (able to trigger biological responses) or bio‐responsive hydrogels (able to respond locally to cell or tissue signals without any external/artificial stimulus). Bioactivity and responsiveness are currently largely explored in order to improve hydrogel interaction with the biological environment in the biomedical field. Thus, innovative hydrogels are able to cross‐talk to cells and respond to the environment, changing their structural properties upon external inputs (*i. e*., temperature, pH, ionic strength, magnetic or electric fields, electromagnetic radiation, biomolecule concentration, etc.). The external stimulus may then promote drug delivery, self‐healing, bonding/nonbonding, pore size modification, viscoelasticity changes, tissue regeneration, etc.

Hydrogels for regenerative medicine applications are currently designed to mimic the complexity of cell/tissue microenvironment, *i. e*. the extracellular matrix (ECM), and most advanced tissue regeneration approaches^[4]^ rely on the strict and active interaction between cells and/or tissues with the materials, in order to stimulate and drive the regenerative process exploiting as much as possible the natural mechanisms.

**Figure 1:**
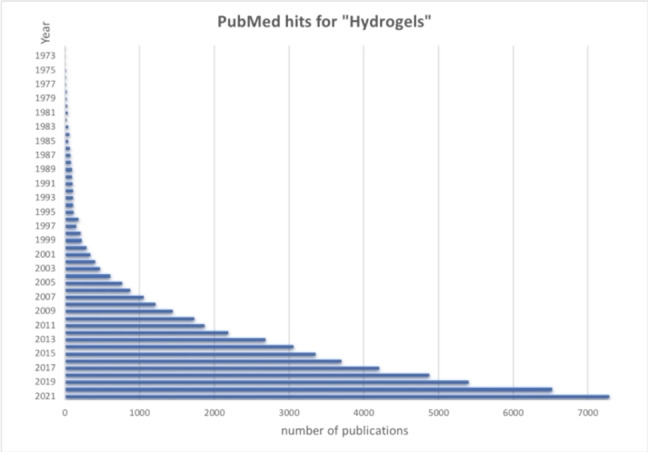
Publication number returned by PubMed for “Hydrogels” in the last 50 years (1972–2021).

**Figure 2:**
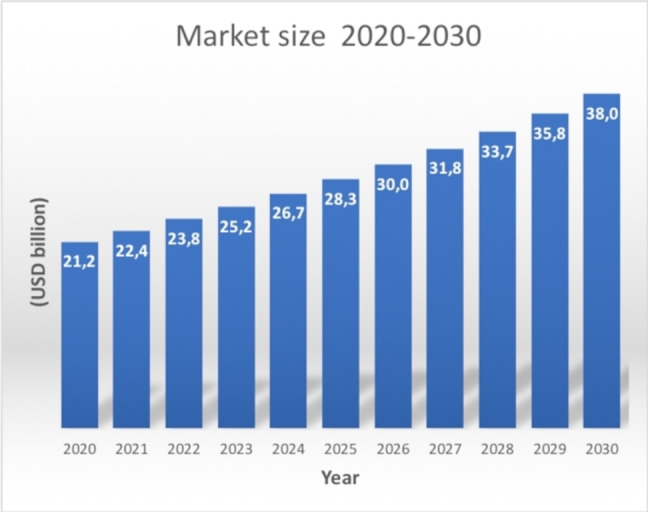
Hydrogel global market size forecast to 2030.

Nowadays, the search for improved hydrogel performances is a very active field both in academia and industry, as confirmed both by the increasing publication number (Figure 1) and market size (Figure 2).

The worldwide market size for hydrogels is expected to reach 40 billion USD by 2030, growing at a CAGR of 6%, from the valued 20 billion USD in 2020 (Figure 2).^[5]^ The market is expected to rise thanks to the use of natural and biodegradable polymers, addressing environmental issues, and sustainability. The market spans several industrial sectors, ranging from pollutant adsorbent materials for water and soil in agriculture, food packaging, cosmetics, separation materials of macromolecules, electronics. However, hydrogels are mostly employed in the biomedical and pharmaceutical field^[6]^ for wound dressing,^[7]^ tissue regeneration,^[8]^ drug delivery,^[9]^ evaporative cooling packaging for therapeutics,^[10]^ contact lenses,^[11]^ personal and hygiene care.^[12]^ Globally, the biomedical sector is expected to drive the market growth in the next few years, with innovative features and new applications.^[13]^ Drug delivery and diagnostics, wound care and tissue regeneration will be crucial market drivers, with the last sector acting as a leader with a 35% whole annual growth rate (CAGR) in 2018–2023.^[14]^ Possible limitations to market expansion are mainly given by sterilisation and production costs.

Hydrogels are composed of polymer macromers that can be either natural, synthetic or a combination of the two. To date the market has been dominated by the synthetic macromers, since they show long employment stability, high water absorbing capacity, high gel strength, on top of being chemically defined and at the same time synthetically flexible. The synthetic hydrogel market is dominated by polyacrylates, polyacrylamides, polyethylene glycol (PEG), polyvinyl pyrrolidone (PVP), polyvinyl alcohol (PVA). On the other hand, the natural hydrogel segment is growing fast, mainly for tissue regeneration and 3D cell culture applications. Natural polymers such as proteins (*i. e*., collagen, gelatin, silk proteins) and polysaccharides (*i. e*., hyaluronic acid, alginate, chitosan) are considered as the most promising hydrogel macromers. Cross‐linking among macromer chains is a crucial issue in determining the final properties (mechanics, chemical and/or biological stability, hydrophilicity, safety and toxicity, biodegradability, stimuli responsiveness) and applications of hydrogels.

Beside cross‐linking, the hydrogel market strongly relies on affordable manufacturing techniques and safety issues. Hydrogels may be manufactured with different morphologies, and several fabrication techniques are available. They are marketed mainly as buttons, impregnated gauzes, sheets, films and matrices; the latter two are the products currently dominating the market, mainly for the biomedical sector. They are used for drug delivery, wound healing, 3D cell culture platforms, soft tissue regeneration, anti‐adhesive membranes, and coating in physiologically interfaced devices. Sheets are clinically applied to the treatment of ulcers, skin burns, wound draining and necrosis, dermatitis.[^6^, ^15^]

Manufacturing techniques affect scalability and reproducibility, and strongly influence market access.^[16]^ Quite often, innovative hydrogel products are based on time‐consuming processes, dealing with viscous solution management, precursor preparation, cross‐linking and washing steps. These steps are frequently manually performed, non‐standardized and with low throughput, raising relevant reproducibility issues to scale‐up and industrial applications.^[17]^ In addition, scale‐up and standardisation need to be flanked by regulatory compliance, when the hydrogel is intended for biomedical use.^[18]^

Most hydrogel manufacturing techniques for large scale productions are commonly divided into two strategies: i) polymerization of hydrophilic monomers in the presence of an initiator and a cross‐linker, or ii) physical or chemical cross‐linking of preformed natural and/or synthetic macromers.^[19]^ In the case of monomer polymerization, different techniques (*i. e*., solution, suspension or bulk polymerization), and initiator (by physical methods such as heat, irradiation by UV‐, gamma‐ or electron beams, or by chemical methods with ammonium peroxodisulphate, benzoyl peroxide, 2,2‐azo‐isobutyronitrile) can be used. The progress in micro‐ and nanofabrication techniques allows the design of hydrogel scaffolds with promising features for regenerative medicine: lithography, 3D printing, electrospinning may help to sum‐up ECM and/or tissue features,^[20]^ however scale‐up is still a challenge.

Manufacturing techniques allow the fabrication of multifunctional materials, and resulting hydrogels are indeed extremely versatile platforms for a plethora of applications. Their morphological, physical, chemical and biological properties may be tuned and combined in order to obtain multifunctional hydrogels for the desired application: self‐healing, responsiveness, reversibility, shear‐thinning, on‐demand actuation, bioactivation with physical and/or chemical stimuli are some of the array of functionalities that can be included in hydrogel design.^[21]^ Selected examples will be considered in the upcoming sections.

Moreover, hydrogel safety is an essential requisite for industrial production. Safety is mainly determined by the starting materials, chemicals/solvents used during the fabrication process, and sterility in case of hydrogel for biomedical applications. Starting materials safety concerns should consider both environmental issues regarding sustainability (renewable resources) and toxicity to the environment, and to living organisms. Safety issues are particularly important in the monomer polymerization approach, often requiring careful washing out of residual monomers, initiators, cross‐linkers, solvents and possible by‐products. When hydrogels are obtained by cross‐linking of pre‐formed macromers, safety issues and purification steps can be reduced, especially when physical cross‐linking is performed (this may not be the case for chemical cross‐linking, *vide infra*); for example, if gamma‐radiation is used as a mean of cross‐linking, concurrent sterilisation does occur. When considering hydrogel for biomedical applications, sterilisation steps come into play for a safe clinical use. This essential and terminal step of the manufacturing process may alter chemical, physical, and biological properties; these modifications are strictly dependent both on hydrogel chemical composition and sterilisation conditions and hence need to be considered specifically for any system.^[22]^

In addition, since biodegradability is a relevant feature for hydrogels in the tissue regeneration field, safety studies should encompass toxicity of degradation products, deriving from hydrogel chemical composition.

Since the biomedical field will be a key player for the hydrogel industrial sector, this review aims at giving chemical and mechanical insights on design criteria that should be taken into account in hydrogel production for biomedical applications. The first part is mainly focussed on chemical aspects of hydrogel design: an introduction on chemical features of polymer macromers and their interaction with the biological environment, and an overview of selected chemical cross‐linking strategies will be first considered; chemistry beyond smart/responsive hydrogels will follow. Since the chemistry of the system dictates the final physical properties, insights of the mechanical properties of hydrogels will be then considered in light of tissue engineering exploitation.

Due to the extensive bibliography about hydrogels, the discussion cannot be exhaustive; selected examples will be considered.

## 2 Macromers chemistry

Macromers found in hydrogels can be of natural or synthetic origin; hydrogels can be assembled with a unique component, or more often are made up with blends of different macromers either synthetic or natural or a mixture of both, ideally giving rise to infinite combination, allowing to explore a huge space of chemical, physical and biological properties suitable for several applications. The chemical composition of macromers and their functional groups define cross‐linking strategies, hydrophilicity/hydrophobicity balance, interaction with the environment (*i. e*., cells, extracellular environment, tissues) and responsiveness to external stimuli. The choice of macromers used for the production of hydrogels for regenerative medicine applications is of particular relevance: they should be suitable to mimic cell native microenvironment (ECM) as much as possible, in order to drive cell adhesion, migration and differentiation to promote tissue repair.[^23^, ^24^, ^25^] Depending on their chemical composition, macromers may have either a structural role (physical properties), a functional role (biological or stimuli responsive properties) or both. For example, macromers may promote biological interactions and cell attachment through passive or active adhesion mechanisms. Functional groups present on the macromers tune material wettability, and surface energy, conferring material hydration properties influencing passive adhesion mechanisms.[^26^, ^27^] Highly hydrophobic materials with low surface energy do not favour cell adhesion, since they do not interact with cell membrane proteins. Likewise, extremely hydrophilic materials or rich in polyelectrolyte groups are highly hydrated with water molecules affording a barrier impairing biomolecule adsorption^[28]^ and cell adhesion.^[29]^ A similar non‐adhesive effect is observed with zwitterionic neutral polyelectrolytes (*i. e*., phosphoryl choline),^[30]^ containing functional groups with opposite charges. It should be noted that mammalian cells are negatively charged,[^31^, ^32^] hence materials with an excess of polyanionic character (*i. e*., carboxylates) have unfavourable effects on cell adhesion, due to electrostatic repulsion. On the other hand, materials with positive charges (*i. e*., amine groups that are protonated to the corresponding ammonium ion at physiological pH), promote cell adhesion and cell proliferation.^[33]^ Active (multi‐step) cell‐adhesion mechanisms may also occur if the material presents suitable mechanical,^[33]^ topographical,[^34^, ^35^] or (bio)chemical signals mimicking the native ECM.^[36]^ After the first non‐bonding interactions, cells cross‐talk with the material through their specific receptors and the material drives cell fate (adhesion, proliferation, differentiation). In the following sections a classification of macromers based on their monomer nature will be given, with a few examples of polymers that have found application.

### 2.1 Macromers from synthetic monomers

Most synthetic macromers lack biochemical cues promoting active cell‐adhesion mechanisms, and depending on their chemical composition also passive cell‐adhesion mechanisms can be hampered. These issues should be carefully considered when designing hydrogels for regenerative medicine applications.

#### 2.1.1 Polyacrylates and polyacrylamides

**Figure 3:**
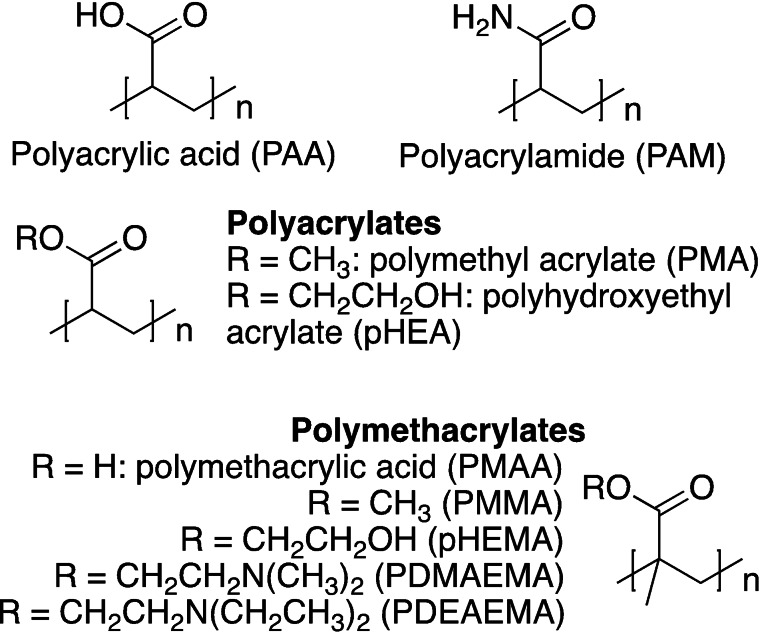
Chemical structure of most common polyacrylic‐based macromers.

Among the most used synthetic polymers for hydrogel fabrication, polyacrylic acids (PAA), polyacrylates (PA) and polyacrylamides (PAM) are driving the market. They are featured with repeating free carboxyl groups, or the corresponding esters and amides, respectively (Figure 3). PAA possesses repeating carboxyl groups that confer highly hydrophilicity and wettability. As observed in the 50’s by Wichterle and Lim, the anionic polyelectrolyte character of polyacrylic acids at physiological pH has unfavourable effects, however control over free carboxyl groups can be exploited for tuning cell interactions^[37]^ and for the preparation of pH‐responsive hydrogels. Currently, several polyacrylic‐based polymers and copolymers (Figure 3) such as poly(methacrylic acid) (PMAA), poly(dimethyl aminoethyl methacrylate) (PDMAEMA), poly(diethyl aminoethyl methacrylate) (PDEAEMA), poly(acryl) amide (PAM) are largely used in the biomedical sector due to their biocompatibility, low cellular uptake and bloodstream stability. In addition, their tunable mechanical and chemical properties make these macromers useful for responsive hydrogels.^[38]^

#### 2.1.2 Polyethylene glycol

**Figure 4:**
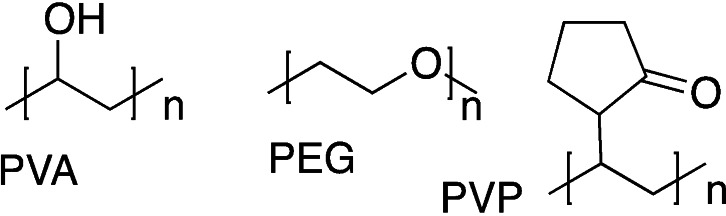
Chemical structure of PEG, PVA and PVP.

Poly(ethylene glycol) (PEG, Figure 4) is a neutral polar and highly hydrophilic synthetic macromer for hydrogel design largely used for biomedical applications, due to its excellent biocompatibility. The presence of extended diethoxy repeating units as hydrogen bond acceptors is responsible for the formation of a water hydration barrier^[39]^ causing cell‐inertness and impairing protein adsorption and cell adhesion. For these reasons, PEG‐based hydrogels were initially proposed as drug delivery platforms or as anti‐adhesive materials.^[40]^ More recently, PEG copolymerization and/or functionalization with adhesive signals such as the integrin binding tripeptide Arg‐Gly‐Asp (RGD)^[41]^ renders this macromer suitable for regenerative medicine applications, and for the design of innovative stimuli responsive hydrogels.^[42]^

#### 2.1.3 Polyvinyl pyrrolidone, and polyvinyl alcohol

Both macromers (Figure 4), being synthetic, do not possess any active interactions with cell receptors, so they need further bioactivation steps if used in biomedical applications.

*Polyvinyl pyrrolidone (PVP)*. PVP is obtained by polymerization of *N*‐vinyl pyrrolidone. Due to its excellent hydrophilicity properties, it was first used as a co‐monomer in pHEMA‐PVP hydrogel for contact lenses; currently, it is usually found in hydrogels as a comacromer.^[43]^

*Polyvinyl alcohol (PVA)*. PVA is obtained by vinyl acetate polymerization, followed by controlled ester hydrolysis to the corresponding polyol. At first, hydrogel properties may be controlled by the degree of deacetylation, allowing to tune polymer water solubility and crystallinity.^[44]^

### 2.2 Macromers from natural monomers: proteins and peptides

Most common proteins used in hydrogels for biomedical applications are of mammalian origin, such as collagen, gelatin, and elastin; non‐mammalian silk proteins are however gaining interest. In general, proteins and peptides offer the advantage of being biocompatible, biodegradable, rich of functional groups that can be exploited for biological interactions and/or chemical and physical cross‐linking or functionalization. However, proteins also have few limitations: being biological derivatives, chemical structural heterogeneity and purity can be key issues, and they may elicit adverse immunological effects.^[44]^ Peptides are commonly obtained by solid phase synthesis (SPPS), thus limiting chemical inhomogeneity.

#### 2.2.1 Collagens and gelatins

Collagens are the most abundant family of proteins in mammals, accounting for 30% in weight of total protein content.^[45]^ Collagens possess specific binding motifs to integrins,^[46]^ thus naturally promote active cell‐adhesion mechanisms; furthermore, collagens are interesting carriers for biochemical cues such as growth factors and cytokines.^[47]^ Because of these properties, collagens are one of the most interesting macromers for hydrogels to be applied in regenerative medicine approaches. It is usually extracted from tissues, thus raising immunological concerns, if not suitably purified from immunogens. Recently, recombinant collagen is facing the biomaterial market, reducing safety concerns, despite high costs.^[48]^ Immunological and cost issues are limited in gelatin. Gelatin is obtained by collagen denaturation, by chemical or physical methods. Gelatin is water soluble above 40 °C, while it gels on temperature decrease. Mechanical features of gelatin are not suitable for tissue engineering applications, hence cross‐linking by chemical or physical methods is needed. Gelatin maintains part of the integrin binding motifs of collagens, thus promoting active cell‐binding mechanisms.

#### 2.2.2 Elastin

Elastin is the main protein responsible for the elastomeric and tensile properties of ECM and many relevant tissues, such as the pulmonary and cardiovascular ones.^[49]^ As such, elastin is a promising macromer for angiogenesis and cardiovascular tissue regeneration.^[50]^ There are still some limitations in the use of elastin as macromer due to tissue extraction procedures, purification and high costs. Recombinant techniques are applied to the production of recombinant human elastin‐like polypeptides, to be used as synthetic mimics of physical and biochemical features of the whole protein.^[51]^

#### 2.2.3 Silk proteins

Silk fibres are produced by worms and spiders and are attractive for hydrogel fabrication in the biomedical field.[^52^, ^53^] The major commercial source of silk fibres is *B. mori*. Silk proteins are biocompatible with human tissues, as witnessed by their use as surgical suture for a long time. Silk fibres are mainly composed of two proteins, silk fibroin (70–80%) and sericin (20–30%). However, fibroin and sericin differ in their amino acids composition, structure, biological and mechanical properties. Fibroin is rich in hydrophobic amino acids of the highly repetitive motif Gly‐Ala‐Gly‐Ala‐Gly‐Ser, forming β‐sheets. Fibroin is largely considered as material for biomedical applications and hydrogel fabrication, since it shows biodegradability, blood compatibility, and cell‐adhesive properties. On the contrary, silk sericin is an adhesive protein rich in hydrophilic amino acids such as aspartic acid, glutamic acid, lysine, serine, and tyrosine; its structure is assembled in β‐sheets and random coils. Sericine, despite being less exploited in the biomedical field, offers distinct biological properties, such as bone mineral phase nucleation capacity (apatite, relevant for bone tissue regeneration), and enhanced adhesion of human skin fibroblasts.^[54]^ The presence of functional groups in amino acid side chains of sericin renders this protein more attractive for chemical cross‐linking, bioactivation and “hybridization” with other macromers.

#### 2.2.4 Polypeptides

As mentioned above, natural macromers may suffer from availability, structural heterogeneity, purification, metabolic instability and safety issues; in order to address these limitations, albeit maintaining the capability to promote biological interactions such as cell adhesion, differentiation or proliferation, several synthetic polypeptides mimicking relevant features of the native proteins have been proposed over years. In many cases, (bio)chemical cues able to improve the biological^[55]^ or mechanical properties^[56]^ of the final hydrogels can easily be introduced. Within this frame, collagen‐like, elastin‐like, silk‐like polypeptides have been produced and used in hydrogels fabrication. Additionally, since the 90 s, self‐assembling peptides of different origin have been proposed for the fabrication of stable scaffolds, including hydrogels.[^57^, ^58^] Peptide‐based hydrogels are able to drive stem cell differentiation, for example for neuronal tissue regeneration.^[59]^

### 2.3 Macromers from natural monomers: carbohydrates

Carbohydrates used as macromers may have different origins (mammalian, marine organisms, plants); regardless of their source, they usually do not possess motifs able to interact with cell‐adhesive proteins (*i. e*., integrins), and may promote cell adhesion through passive mechanisms via suitable functional groups.

#### 2.3.1 Hyaluronic acid

**Figure 5:**
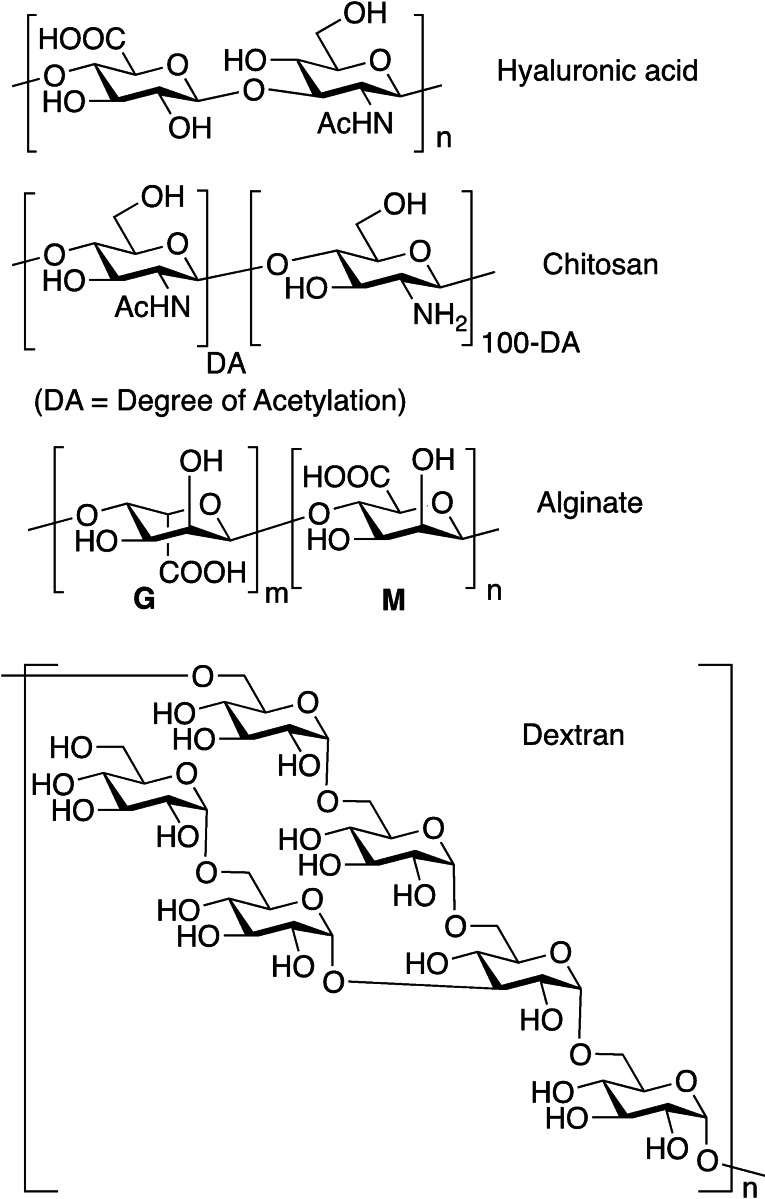
Main polysaccharide structures used in hydrogels.

Hyaluronic acid (HYA) is a polyanionic polysaccharide of mammalian origin constituted by repeating units of β‐1,4‐D‐glucuronic acid and β‐1,3‐*N*‐acetylglucosamine units (Figure 5). It is a non‐sulfated member of the glycosaminoglycan family (GAG), relevant as constituent of the ECM.^[60]^ Its biological function is strictly dependent on the degree of polymerization: low molecular weight oligomers promote endothelial cell migration and angiogenesis, while high molecular weight polymers suppress angiogenesis and show immunosuppressive and anti‐inflammatory properties. HYA is able to interact with cells both with a passive mechanism (*i. e*., physico‐chemical interactions) and active mechanisms through the HYA pericellular coats.[^61^, ^62^]

#### 2.3.2 Chitosan

Chitosan is obtained by controlled deacetylation from chitin, an abundant polysaccharide constituting crustacean's exoskeleton, insect's cuticles, cell wall of fungi and algae. Chitosan is composed of random repeating units of (β1→4) 2‐amino‐2‐deoxy‐D‐glucose (glucosamine) and *N*‐acetyl‐2‐amino‐2‐deoxy‐D‐glucose (*N*‐acetyl glucosamine), (Figure 5). Chitosan, insoluble in water above pH 6.5, is a biocompatible, biodegradable, non‐toxic polymer useful as macromer for hydrogel preparation.^[63]^ Its cell‐adhesive properties are controversial.^[64]^

#### 2.3.3 Alginate

Alginate is a polyanionic marine polysaccharide derived from brown algae and constituted by repeating (β1→4) α‐L‐guluronic acid (G) and its D‐mannuronic acid (M) epimer (Figure 5). The sequence of these monomers (MM, GG and GM blocks), the M/G monomeric ratios as well as the polymer molecular weight are strictly dependent on the origin and production process. As other polysaccharides, alginates are biocompatible, however they poorly interact with biological systems (lack of cell‐adhesive motifs and protein adsorption capabilities). Upon suitable modifications or in combination with other (bio)molecules, alginates become interesting macromers for hydrogel preparation towards tissue engineering applications.^[65]^

#### 2.3.4 Dextran

Dextran is a branched neutral polysaccharide of microbial origin, constituted by a α‐1,6 polyglucoside backbone, with α‐1,3 branches (Figure 5). Dextran is biocompatible and nontoxic and its main use is as an antithrombotic agent. In recent years dextran is gaining interest in hydrogel design for tissue regeneration applications and for drug delivery, due to its low toxicity and biodegradation by dextranases, present in the large intestine. However, as in the case of alginate, dextran does not possess the ability to interact with biological systems, with no protein absorption ability and lack of cell‐adhesive motifs.[^66^, ^67^]

#### 2.3.5 Pectin

**Figure 6:**
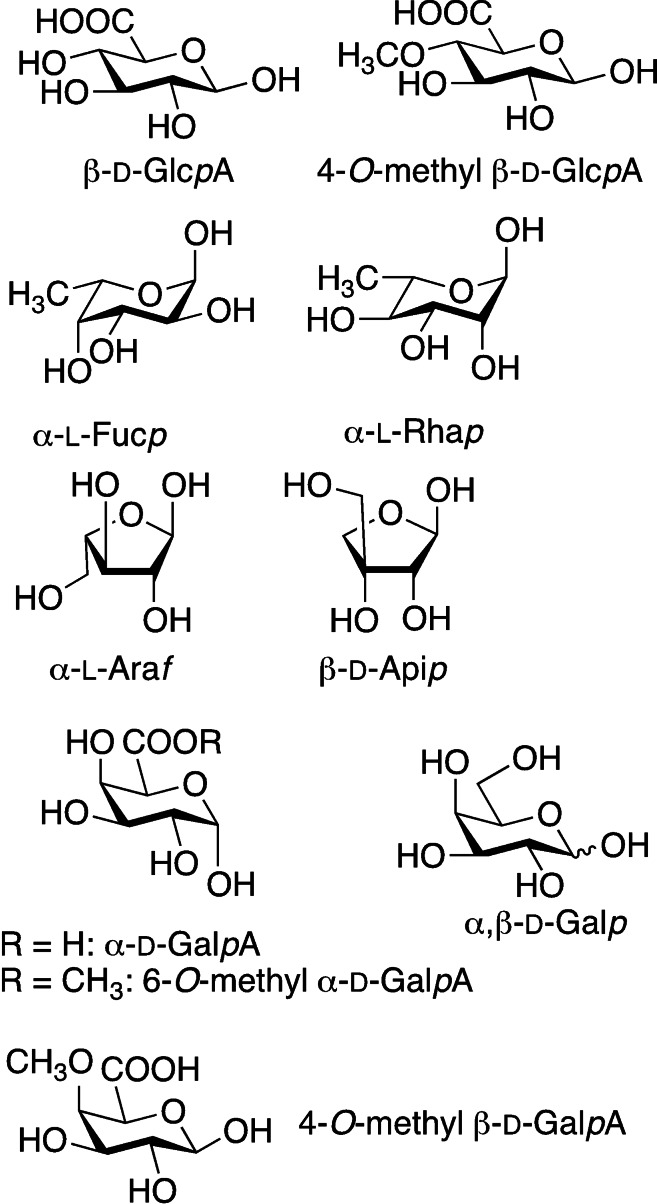
Main monosaccharide units composing pectins.

Pectin is a polyanionic branched polysaccharide as well, found in the cell walls of most plants, and obtained from different fruity sources. It has a complex structure mainly based on three polysaccharide domains, homogalacturonan (HGA), rhamnogalacturonan‐I (RG‐I) and rhamnogalacturonan‐II (RG‐II), built up with furanose and pyranose monosaccharides (Figure 6). As in the case of dextran, pectins are biocompatible, however they lack bioactivity and for this reason they entered the tissue regeneration and hydrogel sector only recently.^[68]^

### 2.4 Macromers from natural monomers: polynucleotides

Polynucleotides are attracting great attention as innovative materials and hydrogels, both as structural and functional macromers. In particular, polynucleotides can be easily synthesised with natural and non‐natural nucleotide monomers that naturally possess a hydrophilic polyanionic structure, together with biocompatibility, controlled biodegradability, and metabolic stability against proteases. Polynucleotides show higher versatility in hydrogel production than polysaccharides and proteins.[^69^, ^70^]

## 3 Macromer cross‐linking chemistry

**Figure 7:**
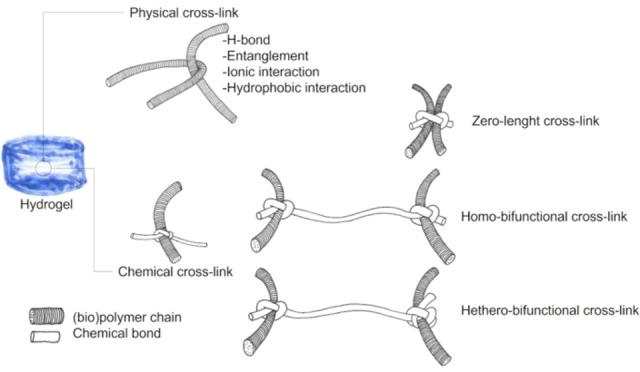
Physical or chemical cross‐linking strategies (only a selection of methods has been depicted).

In order to obtain hydrogels, cross‐linking among macromers is usually needed; cross‐linking is a key factor in determining the stability/degradation properties in water and biological environment, viscoelastic behaviour, stimuli responsiveness. Cross‐linking can be obtained by chemical or physical methods (Figure 7).

Physical cross‐linked hydrogels, often referred to as “reversible” hydrogels, are based on molecular entanglements, and/or secondary forces including H‐bonds, ionic interactions, hydrophobic forces, crystallisation, and ligand/receptor interactions. Physical hydrogels are limited by their higher instability in the physiological environment.

In chemical cross‐linked hydrogels, macromers are linked through covalent bonds, affording higher chemical, metabolic, and mechanical stability. However, their main downsides are limited biodegradation and toxicity of the reagents/solvents used in cross‐linking strategies. Underestimation of toxicity of reagents and byproducts that frequently remain entrapped within the gel is an issue; in addition, the chemical reactivity of the system is complex, and no detailed description of competing reactions is given. This section is focused on chemical cross‐linking, and a detailed description of mechanisms, possible competing reactions and reagents/byproduct toxicity is mentioned.

Chemical cross‐linking relies on the presence of functional groups able to react upon activation or by the addition of suitable cross‐linker (macro)molecules with complementary reactivity. Different chemistries have been proposed over years, including the so‐called click chemistry strategies exploiting for example Schiff base formation, Michael additions, Huisgen‐type and Diels‐Alder cycloadditions. Click reactions may occur in a biocompatible environment, however they are limited by the need of specific functionalities that are usually absent in macromers of natural origin, and barely present in low cost commercial macromers. While the specific cross‐linking click reaction may guarantee biocompatibility, this cannot be the case in macromer derivatization with the required functionalities. On the other hand, many polymeric macromers, either natural or synthetic, possess amino, carboxyl or hydroxyl groups that can be directly exploited for cross‐linking, without further derivatization. In the next paragraphs a selection of chemical cross‐linking strategies will be detailed.

### 3.1 “Conventional” cross‐linking chemistry

**Figure 8:**
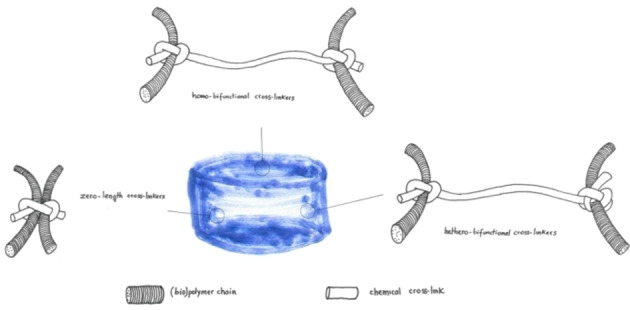
Schematic view of zero‐length, homobifunctional and heterobifunctional cross‐linking.

“Conventional” cross‐linkings are featured with straightforward application, with a reduced number of synthetic steps, lower costs, based on functional groups already in place in macromer precursors, and thus frequently used in hydrogel fabrication. Some strategies form direct chemical bonds between macromer functional groups, and can be considered as zero‐length crosslinking (Figure 8). In other cases, a homobifunctional or heterobifunctional cross‐linker (macro)molecule is needed. Depending on the cross‐linker length, stronger or looser gels can be obtained.

**Scheme 1:**
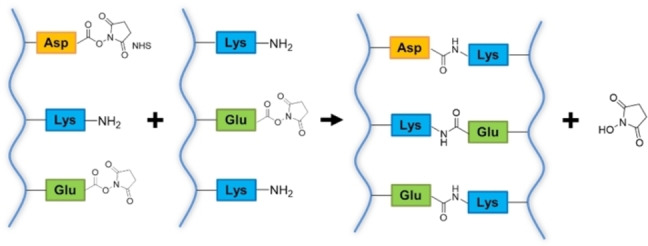
Schematic outline of zero‐length cross‐linking between proteins, mediated by EDC/NHS activation.

***Amide bond formation***. Macromers containing −COOH and −NH_2_ groups (*i. e*., proteins, PEG, polyacrylate derivatives) can be cross‐linked with a zero‐length cross‐linking strategy involving the activation of the carboxyl groups (*i. e*., Asp or Glu in proteins) to allow the direct reaction with amino groups (*i. e*., Lys in proteins). A covalent amide bond is formed between the two reacting groups and no potentially toxic cross‐linker moieties are grafted to the hydrogel matrix (Scheme 1).^[71]^

**Scheme 2:**
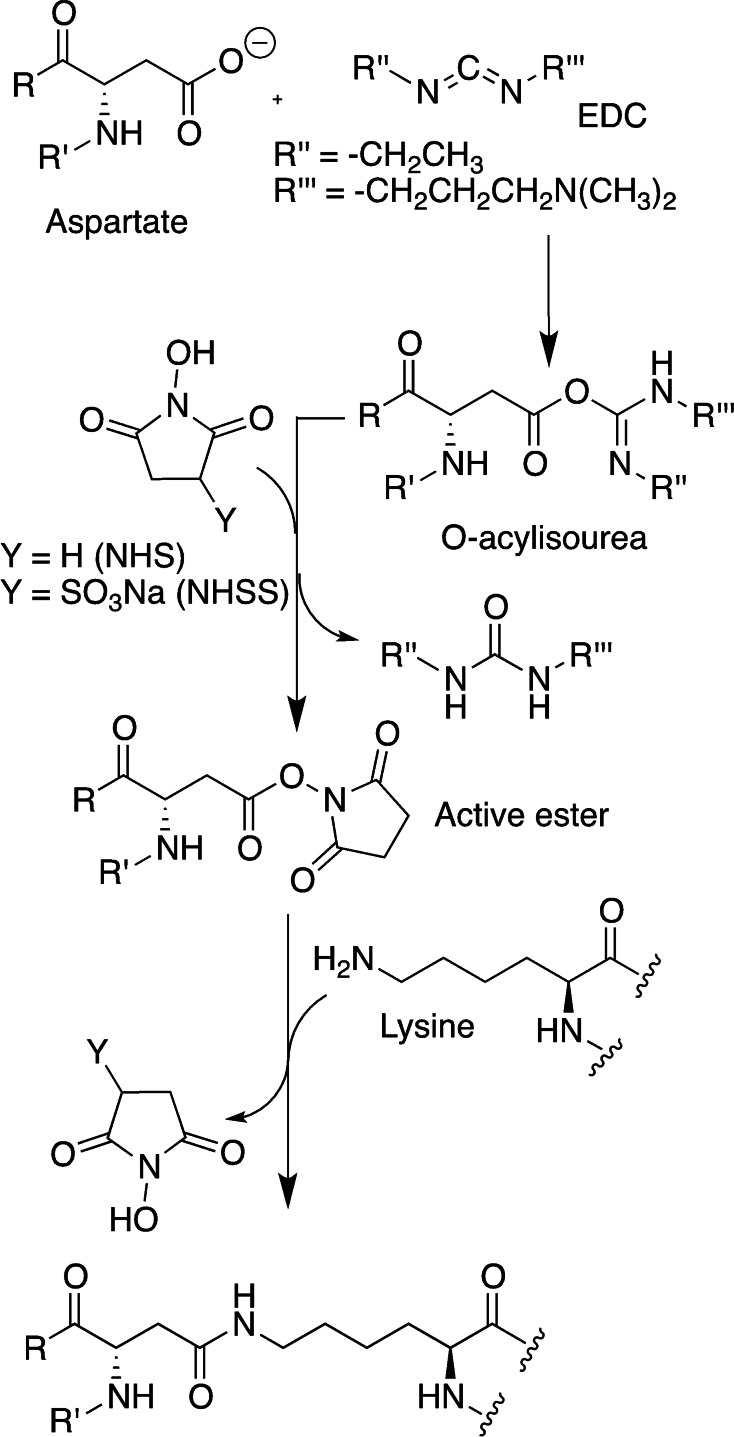
Reaction mechanism for isopeptide bond formation between carboxyl group of aspartate and amino group of lysine in proteins, mediated by EDC and NHS (or NHSS).

1‐Ethyl‐3‐(3‐dimethylaminopropyl)‐carbodiimide (EDC)/N‐hydroxysuccinimide (NHS) is one of the most commonly used strategies for zero‐length cross‐linking. EDC forms an *O*‐acylisourea adduct upon reacting with carboxyl groups, which can in turn give a nucleophilic acyl substitution with amino groups affording an amide bond. However, in the presence of water the hydrolysis of the *O*‐acylisourea is prevalent, leading to the release of the original carboxyl group and an *N*‐substituted urea by‐product.^[72]^ This reaction is fast, the estimated rate constant at pH 4.5 is of the order of 2–3 s^−1^, and completely takes over the desired cross‐linking reaction.^[73]^ To solve this issue, an adduct more stable than *O*‐acylisourea must be formed, and NHS or *N*‐hydroxysulfosuccinimide (NHSS) were found to be good reactants to form active esters.^[72]^ After the formation of the *O*‐acylisourea adduct, the hydroxyl group of NHS acts as a nucleophile and attacks the carboxyl group, leading to the release of *N*‐substituted urea as a byproduct. The *N*‐succinimidyl ester obtained is relatively stable, and can give nucleophilic substitution reaction with amino groups without being hydrolysed, forming the amide bond (Scheme 2).^[72]^

**Figure 9:**
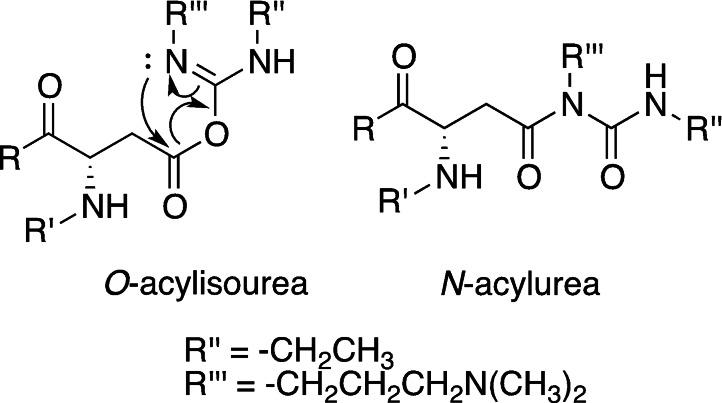
Isomerisation of the O‐acylisourea to an *N*‐acylurea stable derivative (tetrahedral intermediate deriving from the nitrogen attack to the carbonyl of the *O*‐acylisourea is omitted).

Zero‐length cross‐linking is advantageous compared to other forms of cross‐linking, because no reverse reaction and release of the cross‐linking moieties can occur upon hydrogel degradation.^[74]^ However, the excess of the coupling agent and its by‐products are released during the reaction and are not incorporated in the material, causing possible cytotoxicity. EDC is the most used carbodiimide for cross‐linking purposes, because it is known for being non‐toxic and biocompatible if the obtained hydrogel is thoroughly washed, it is a mild reagent that doesn't denature protein, and it is water soluble.[^71^, ^75^] For example, gelatin cross‐linked by EDC/NHS displays a higher degradation temperature, tensile strength, elongation at breaking point and stiffness. Moreover, the swelling capacity is comparable to that of non‐crosslinked gelatin.[^71^, ^74^] Despite the aforementioned advantages, some disadvantages emerge mainly for side‐reaction and by‐product formation. Cysteine and tyrosine residues in proteins can react with EDC forming almost irreversible sulphur‐carbon or oxygen‐carbon single bonds, while histidine residues can accelerate the hydrolysis of the NHS‐activated ester.^[76]^ Another possible side reaction is the isomerisation of the *O*‐acylisourea derivative to the more stable and unreactive *N*‐acylurea (Figure 9), even though this modification seems to be limited to residues in an hydrophobic environment.^[72]^ This side reaction caps carboxyl groups, limiting cross‐linking.

Regarding the cytotoxicity of the final material, *in vitro* assays on human and murine fibroblast and human keratinocytes were carried out, however the results diverge from each other. It has been reported that 50 mM EDC treated collagen does not perturb the cellular monolayer, and no lysis or morphological changes were observed.^[77]^ Other researchers claim, on the contrary, that 10 mM and 50 mM EDC concentrations drastically lower the density and the organisation of cells, making them unable to form a consistent epidermal barrier. As a consequence, the mechanical properties of the scaffold got worse compared to the one reported for the acellular material.^[78]^ It is commonly acknowledged that these discrepancies can be explained by different methods of preparation and washing of the residual coupling agents and by‐products.^[77]^ Amongst by‐products of EDC cross‐linking, *N*‐substituted urea deserve mention, because studies suggest that concentration of urea above 5 mM can cause oxidative stress in kidney cell lines, leading to DNA damage and carbonylation of proteins.^[79]^ Moreover, it can spontaneously form the cyanate ion, a uremic toxin that accumulates after kidney injuries.^[80]^ Therefore, further evaluation of biocompatibility of EDC cross‐linked hydrogels is required. Amide bond formation among amino acid side chains in proteins raises an additional concern: Asp and Glu residues, being involved in cross‐linking, are not available anymore as adhesive RGD motifs recognised by integrins, namely heterodimeric cell receptors that mediate cell‐cell and cell‐ECM interactions.^[46]^

***Natural cross‐linkers***. Natural dicarboxylic (*i. e*., succinic acid, glutaric acid, adipic acid), and tricarboxylic acids (*i. e*., citric acid) are di‐ and tri‐ functional compounds that can be exploited as homofunctional crosslinkers complementary to −NH_2_ or −OH groups. However, the condensation reaction should be promoted with suitable coupling agents in order to be effective (*i. e*., the previously cited EDC/NHS). On the other hand, a commonly used natural cross‐linker is genipin,^[81]^ able to react with nitrogen nucleophiles, without any need of pre‐activation/derivatization.

**Scheme 3:**
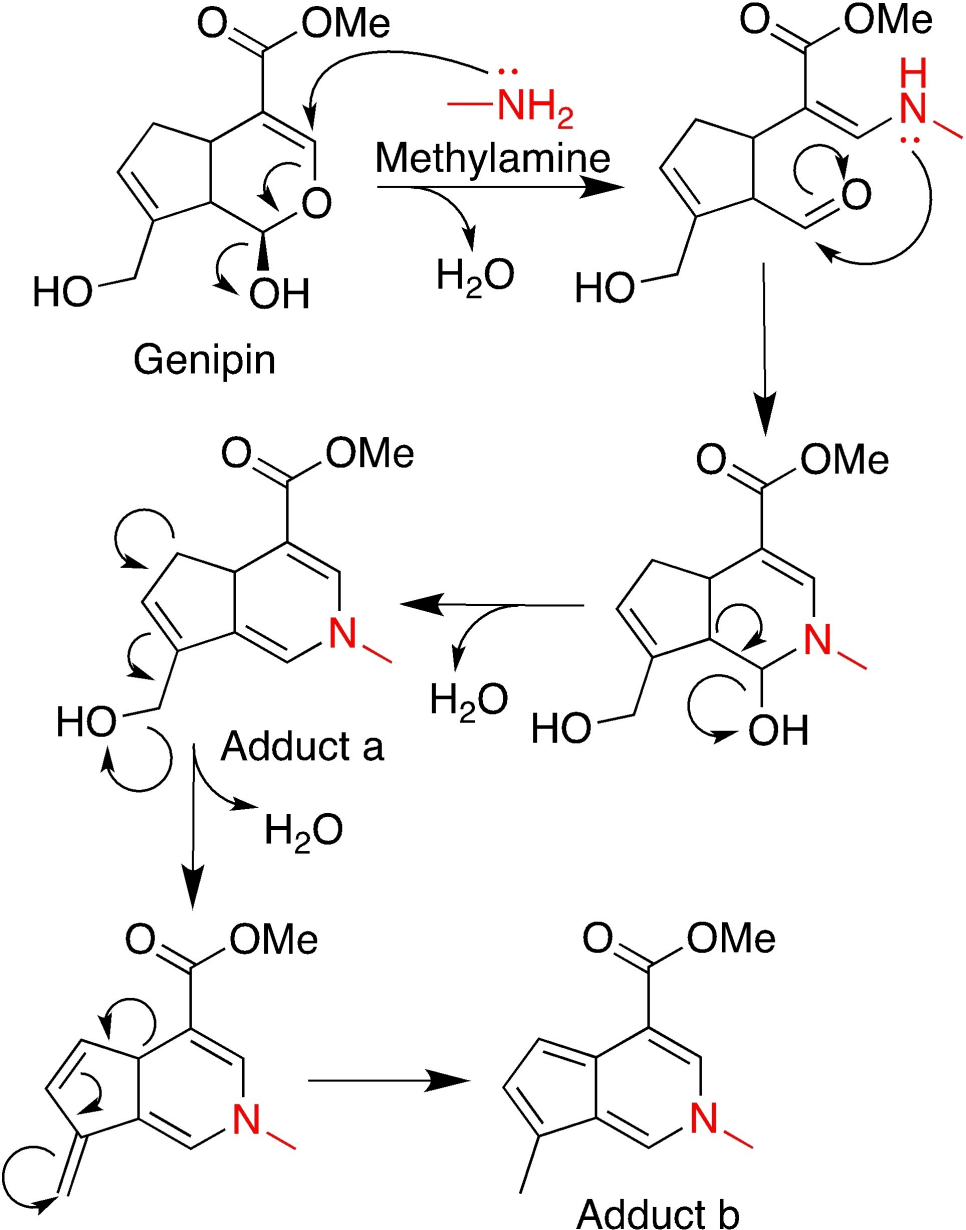
Reaction mechanism for the formation of genipin‐derived adducts **a** and **b** upon reaction with methylamine. Only one of the possible rearrangements of adduct **a** into adduct **b** is shown.

Genipin is obtained by enzymatic hydrolysis of geniposide, a iridoid glycoside extracted from *Gardenia jasminoides Ellis* fruits.^[82]^ This aglycone derivative has been widely used as an antiphlogistic and cholagogues medicine, as well as a food dye due to its capability to form dark‐blue pigments when reacted with amino acids.^[83]^ This ability to form stable covalent bonds with protein amino acids such as lysine, hydroxylysine and arginine makes genipin suitable for homo‐bifunctional cross‐linking and hydrogels preparation.^[82]^ Although the exact cross‐linking mechanism has not been elucidated yet, a hypothesis can be made studying the reaction between genipin and methylamine, the simplest primary amine (Scheme 3). The amine group of methylamine acts as a nucleophile and attacks the C‐3 olefinic carbon of genipin, leading to the opening of the dihydropyran ring and formation of an aldehyde group upon loss of a water molecule. The newly formed secondary amine attacks the aldehyde group, regenerating a six‐member ring with the loss of a second water molecule (adduct a). Further rearrangements may occur, leading to the loss of the primary hydroxyl group of the original genipin, affording adduct b (Scheme 3).[^83^, ^84^]

**Figure 10:**
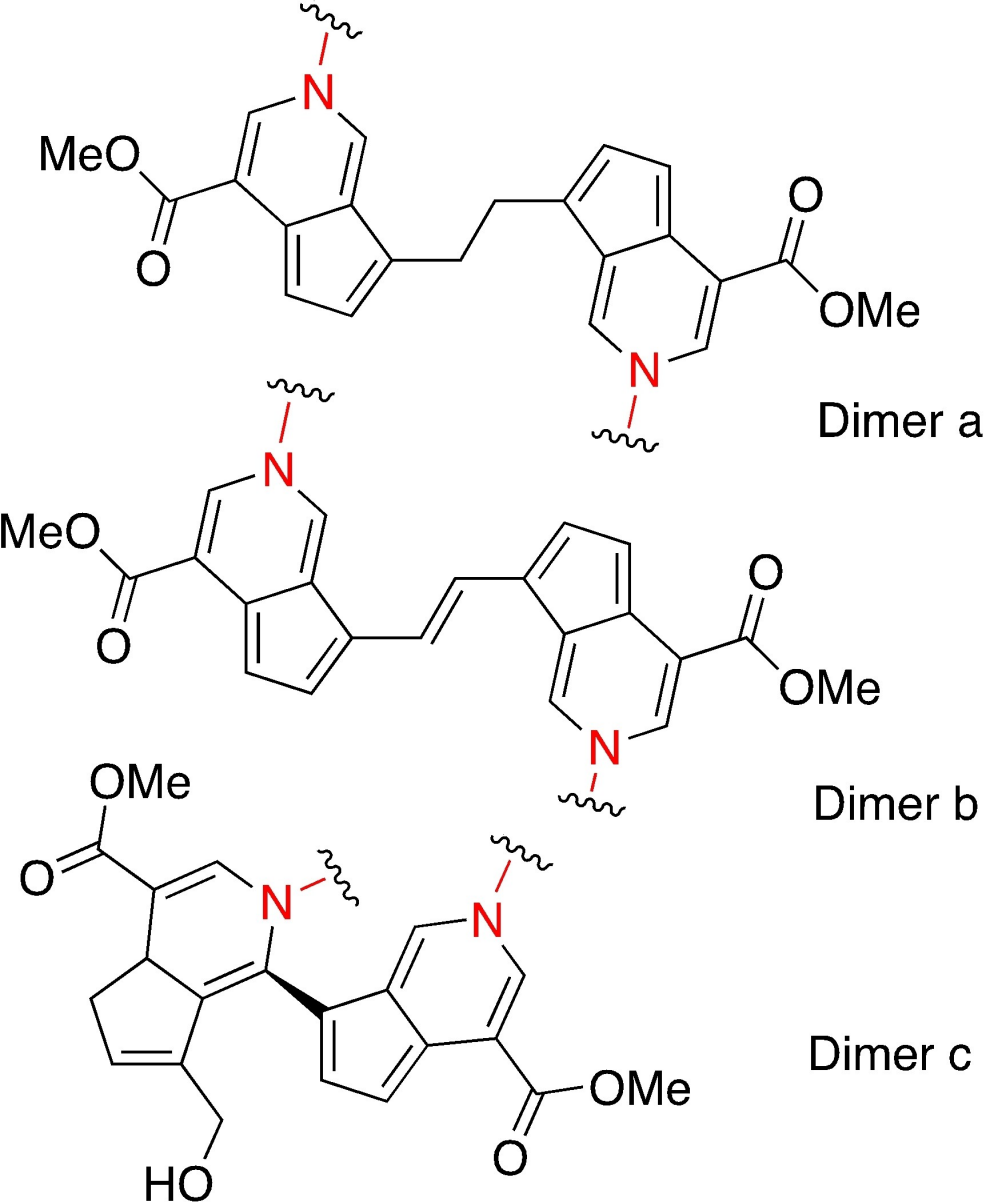
Possible dimerization products derived from genipin adducts obtained by reactions with primary amines (described in Scheme 3 for methyl amine).

These nitrogen iridoids can also form upon reaction with primary amino groups in proteins: the obtained adducts (Adduct a and Adduct b, Scheme 3) can dimerize in different ways, presumably with a radical reaction, leading to cross‐linking (Figure 10). Trimeric and tetrameric structures were also found.^[85]^

Nevertheless, the exact cross‐linking products are difficult to be fully identified, because of the different combinations of monomers and reactions involved.^[86]^ Being a natural compound used in traditional Chinese medicine, genipin attracted great attention for cross‐linking, because it could replace the toxic homo‐bifunctional crosslinker glutaraldehyde (*vide infra*).^[84]^

Many studies confirmed this hypothesis: cytotoxicity tests on human fibroblasts or dermal mesenchymal cells show that cell density in medium supplemented with genipin was much greater than those supplemented with glutaraldehyde.[^82^, ^87^] The lethal concentration of genipin for fibroblasts was estimated at 10 mM,^[88]^ although murine and canine models showed an inflammatory response by the host.[^89^, ^90^] The thermal stability, mechanical properties and resistance to enzymatic degradation of genipin‐cross linked hydrogels are comparable to glutaraldehyde fixed ones.[^82^, ^84^] However, further *in vivo* studies are necessary to assess toxicity of genipin‐cross linked materials. Genipin shows some disadvantages due to low availability and high costs.^[91]^ Moreover, the application of genipin in regenerative medicine is limited by the coloration caused by the reaction with primary amines, which produces a blue pigment that can not be used for transparent tissues such as cornea.^[92]^

### 3.2 Click cross‐linking chemistry

Cross‐linking can be performed based on click chemistry strategies, that is chemical reactions occurring in mild conditions, in water as the solvent (*i. e*., limiting the use of toxic solvents), with high chemoselection and high yields. In many cases, biocompatibility and toxicity concerns of “conventional” cross‐linking are alleviated. Because of their chemoselectivity and bioorthogonality, they can be used to synthesise injectable hydrogels.^[93]^

#### 3.2.1 Schiff base chemistry

**Scheme 4:**
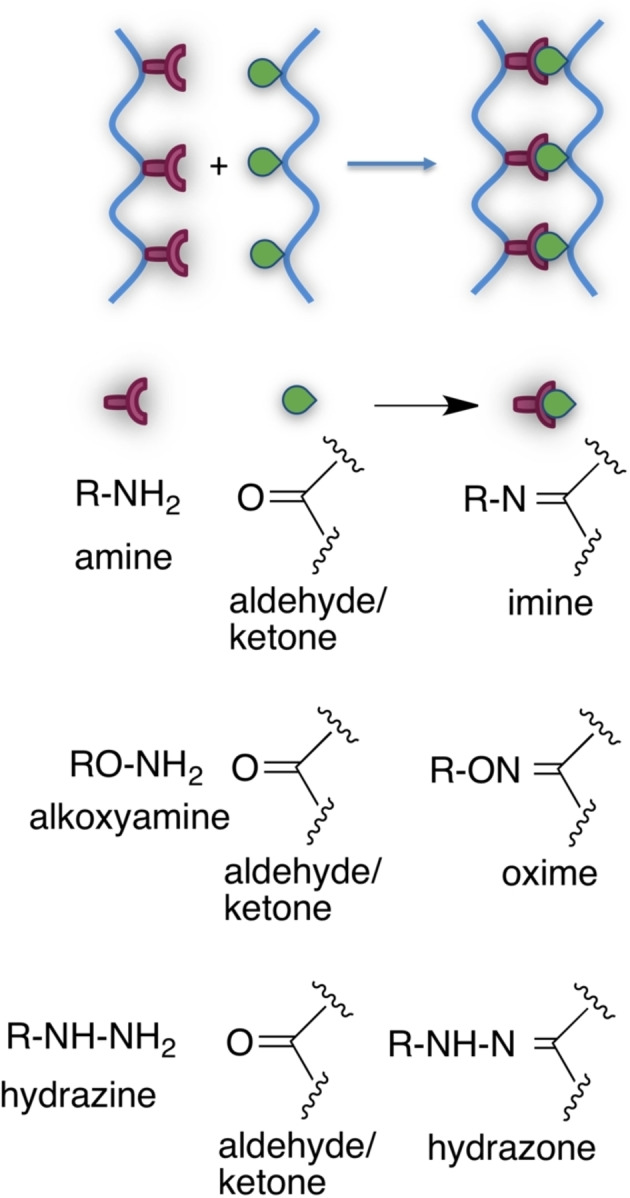
Schiff base cross‐links for hydrogel preparation.

Schiff base cross‐linking is one common and long‐dating click strategy for cross‐linking.^[94]^ The reaction involves the attack of −NH_2_ group of amines, alkoxyamines, hydrazines, to the carbonyl carbon of aldehydes and ketones to the corresponding imines, oximes, hydrazone^[95]^ derivatives containing a C=N double bond (Scheme 4). The stability of cross‐linking depends on the −NH_2_ substituents, usually being oximes and hydrazones more stable than imines. Among imines, stability can be modulated by the introduction of nitrogen substituents able to stabilise the double bond, for example with conjugated aryl groups. Generally, suitable macromer modification is needed to introduce either the nucleophilic moiety or the carbonyl group. When macromers are proteins and saccharides, the mutually reactive functional groups are already in place (amine and aldehyde groups); moreover, the imine bond can be reduced to the more stable amine.^[96]^ However, primary amine‐containing macromers are directly crosslinked with difunctional dialdehyde, such as glutaraldehyde.

**Scheme 5:**
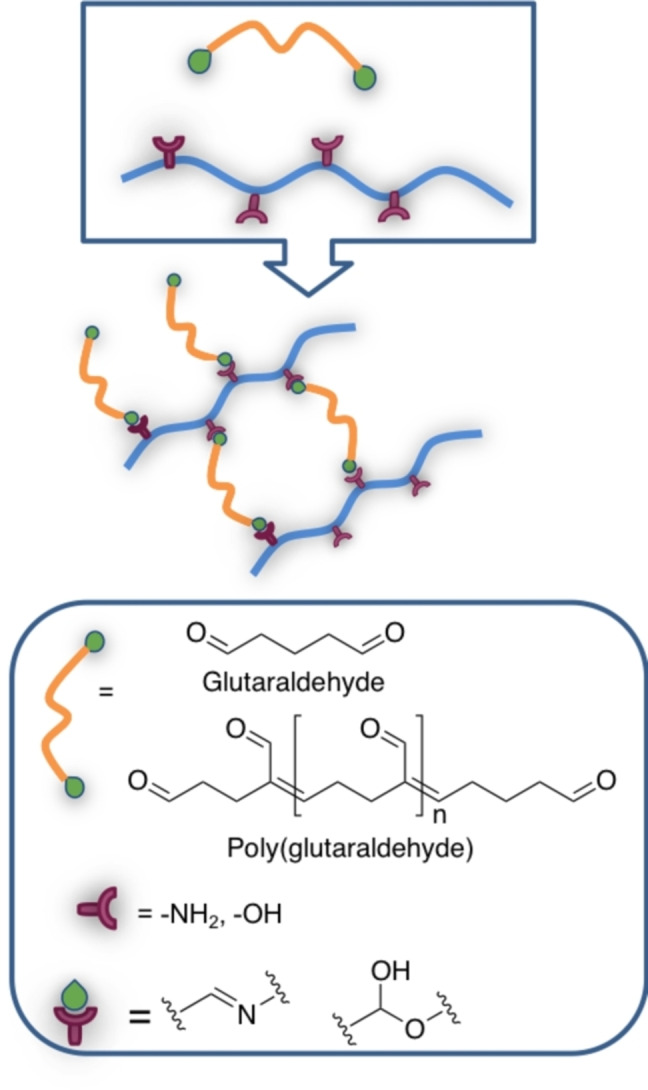
Glutaraldehyde cross‐linking products.

*Glutaraldehyde*. Glutaraldehyde is one of the most widely used homobifunctional cross‐linker, due to its low cost, commercial availability, straightforward reactivity and capability to react directly with proteins and more generally with amino‐containing macromers, improving their thermal, mechanical, and water‐sensitive properties (Scheme 5).^[97]^

**Scheme 6:**
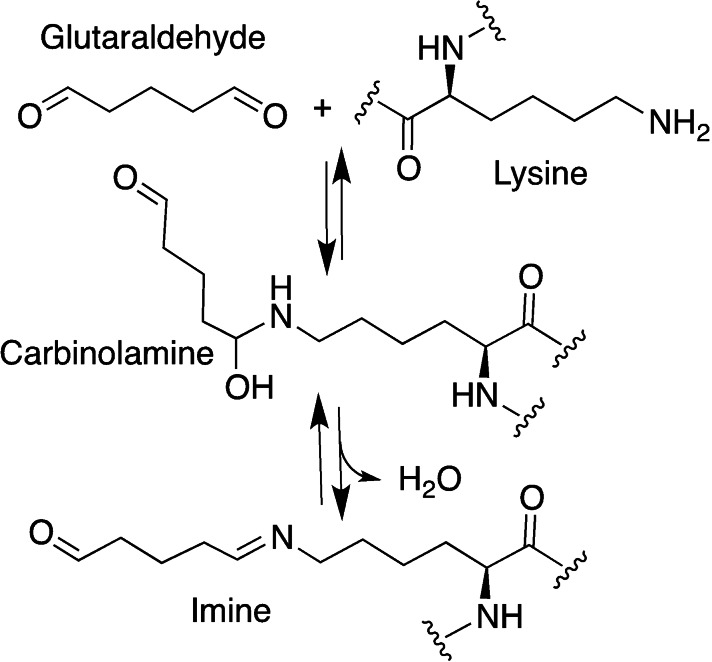
Reaction steps between amino group (i. e. lysine in proteins) and carbonyl group of glutaraldehyde to form the Schiff's base; for crosslinking the reaction occurs on both carbonyls.

Considering polyfunctional proteins, at alkaline pH (11) the ϵ‐amino groups of lysine and hydroxylysine, as well as the amino groups of N‐terminal amino acids are deprotonated, and therefore can act as nucleophiles for the addition to the carbonyl group of the aldehyde to form a carbinolamine; this intermediate is unstable, and the loss of a water molecule yield the conjugated Schiff bases (Scheme 6).^[98]^

The pH value of the medium dictates the cross‐linking mechanism, as a consequence of the different protonation states of functional groups present in the system (*i. e*., amino acids side chains in proteins).

**Scheme 7:**
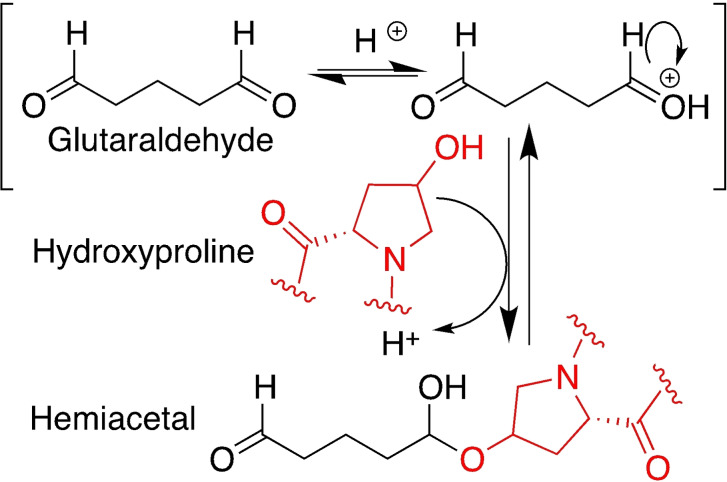
Reaction steps between hydroxyl groups and carbonyl groups of glutaraldehyde to form the hemiacetal (exemplified for the group of hydroxyproline, abundant in collagen); for crosslinking the reaction occurs on both carbonyls.

At acidic pH (4.5), the carbonyl group is activated as the corresponding oxonium ion and the amino groups are mostly protonated and positively charged, therefore they can't give nucleophilic addition. In this case, the hydroxyl groups of serine, threonine and other hydroxylated amino acids (*i. e*., hydroxylysine and hydroxyproline) are competing nucleophiles for the addition at the carbonyl group, affording the corresponding hemiacetal (Scheme 7).^[98]^

**Scheme 8:**
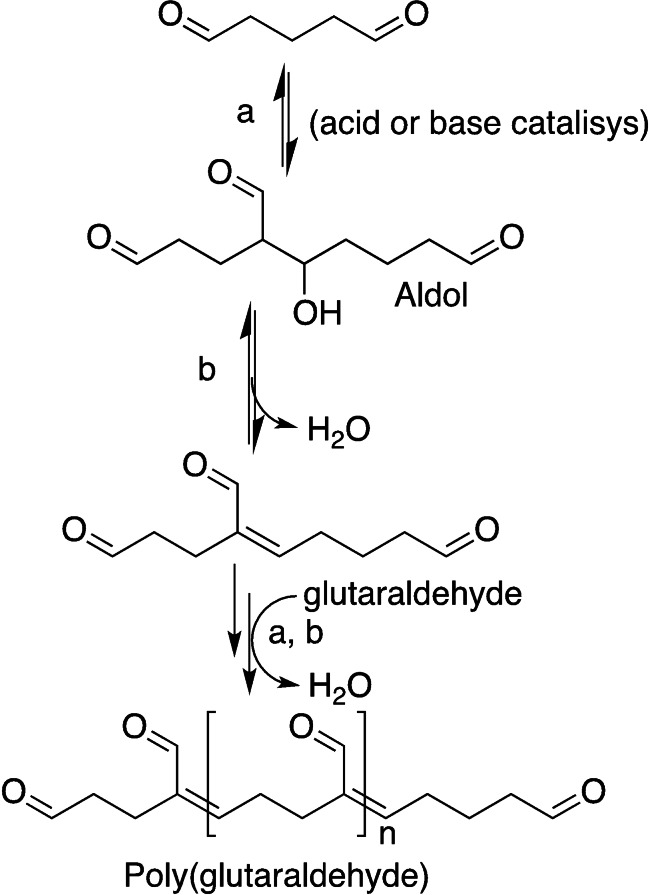
Base‐ or acid‐catalysed aldol condensation among glutaraldehyde molecules.

A competing aldol condensation can occur between two glutaraldehyde molecules, both acid‐ or base‐catalysed, forming longer and branched carbonyl cross‐linkers (Scheme 8).^[98]^ Mechanical properties and thermal stability of the material were especially enhanced at pH 4.5, establishing it as the optimum value for glutaraldehyde mediated cross‐linking.^[99]^

Despite the mentioned advantages of glutaraldehyde, its cytotoxicity can not be overlooked. It has been reported that limiting the unreacted glutaraldehyde with high‐percentage cross‐linking and accurate rinsing with water is sufficient to not hinder cell viability.[^100^, ^101^] However, studies on fibroblast proliferation seeded on the crosslinked gelatin show significant initial inhibition of growth, even at a concentration of 0.6 μg/mL.[^79^, ^102^] Regarding the tissue‐regenerative capacity of the material, dermal sheep collagen and heart or prosthetic valves treated with glutaraldehyde were implanted in rats, and after six weeks the structures were completely calcified and active fibroblast were sporadically present. After fifteen weeks all of the cross‐linked implanted collagen was resorbed and hardly any new collagen formed.[^103^, ^104^] An hypothesis is that calcium in the human body reacts with the aldehyde group of glutaraldehyde, and this complex then combines with the pericardial tissue.^[84]^

#### 3.2.2 Thiol‐chemistry

Thiol groups are frequently encountered in proteins, being the side chain group of cysteine residues, and offer already in place functionalities for cross‐linking. Thiol groups may react with radical or heterolytic reaction mechanisms; regardless of the reaction type, thiol groups have gained interest in recent years in click chemistry approaches for hydrogel network formation.^[105]^

**Scheme 9:**
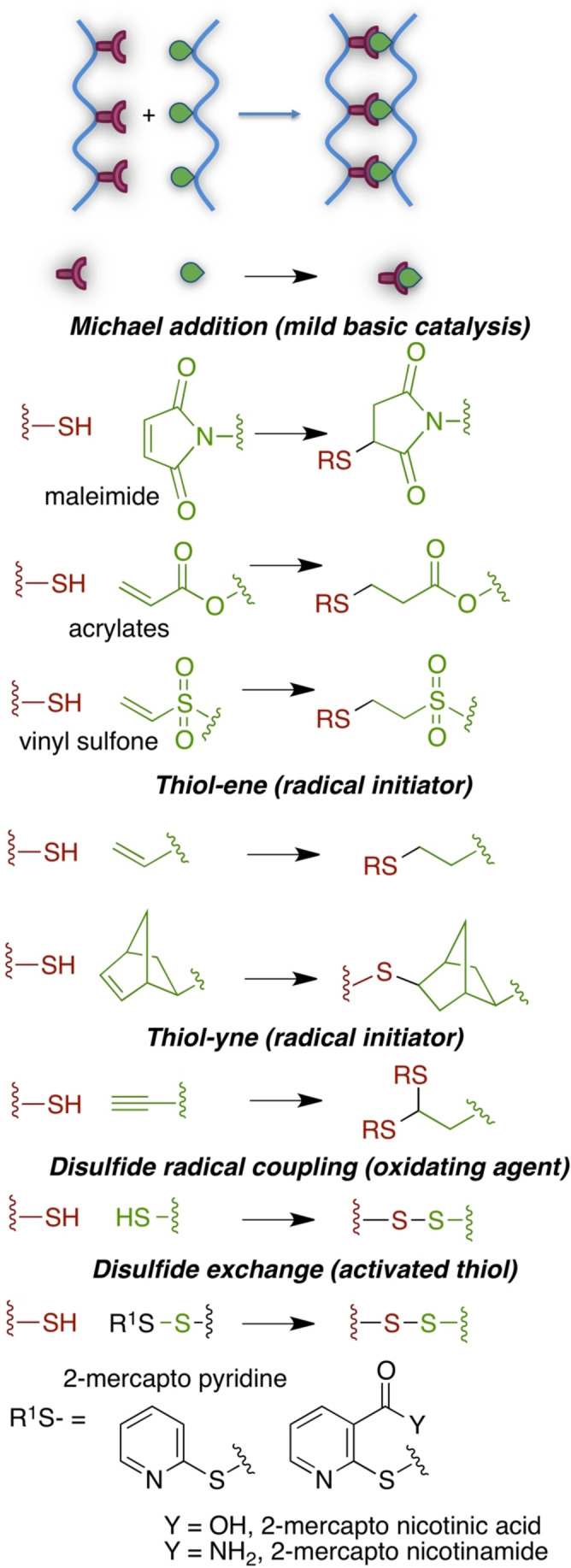
Main thiol‐based click reactions for hydrogel preparation.

In the heterolytic mechanisms, thiol groups act as nucleophiles, for example in Michael additions to electron‐poor double bonds in maleimides, vinyl sulfones, and acrylates, or in thiol disulfide exchange reactions (Scheme 9). Michael addition are considered biocompatible click reactions, however they need suitable derivatization of macromers with Michael acceptor, and eventually with the thiol Michael donor if not already in place (for example, despite collagen being a protein, it lacks cysteine residues).[^96^, ^106^] As for thiol‐disulfide exchange reactions, usually they are performed using an activated disulfide, such as pyridyl‐disulfide, which poses toxicity problems due to 2‐mercaptopyridine byproduct, or more recently mercaptonicotinic acid or the corresponding amide (Scheme 9).^[107]^

**Scheme 10:**
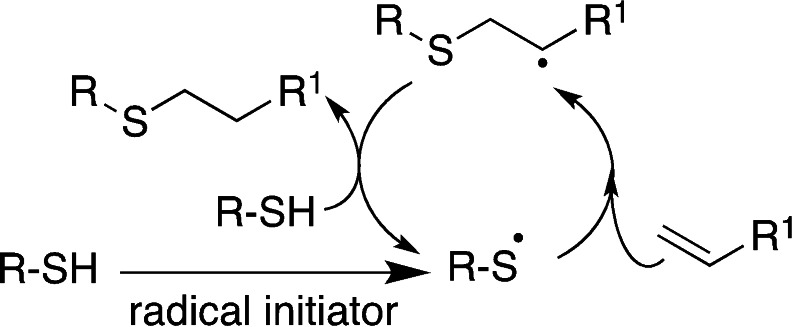
Radical mechanism for the thiol‐ene reaction.

Radical mechanism (exemplified in thiol‐ene reaction in Scheme 10) is involved in oxidative disulfide bond formation, in the click thiol‐ene and thiol‐yne reactions (Scheme 9). Radical thiol‐ene and thiol‐yne reactions need a thermal or photoinitiator, hence toxicity issues should be carefully considered. On the contrary, Michael addition is promoted by a mild basic environment, thus offering great advantages for biomedical applications.

#### 3.2.3 Azide‐alkyne cycloaddition (AAC)

**Scheme 11:**
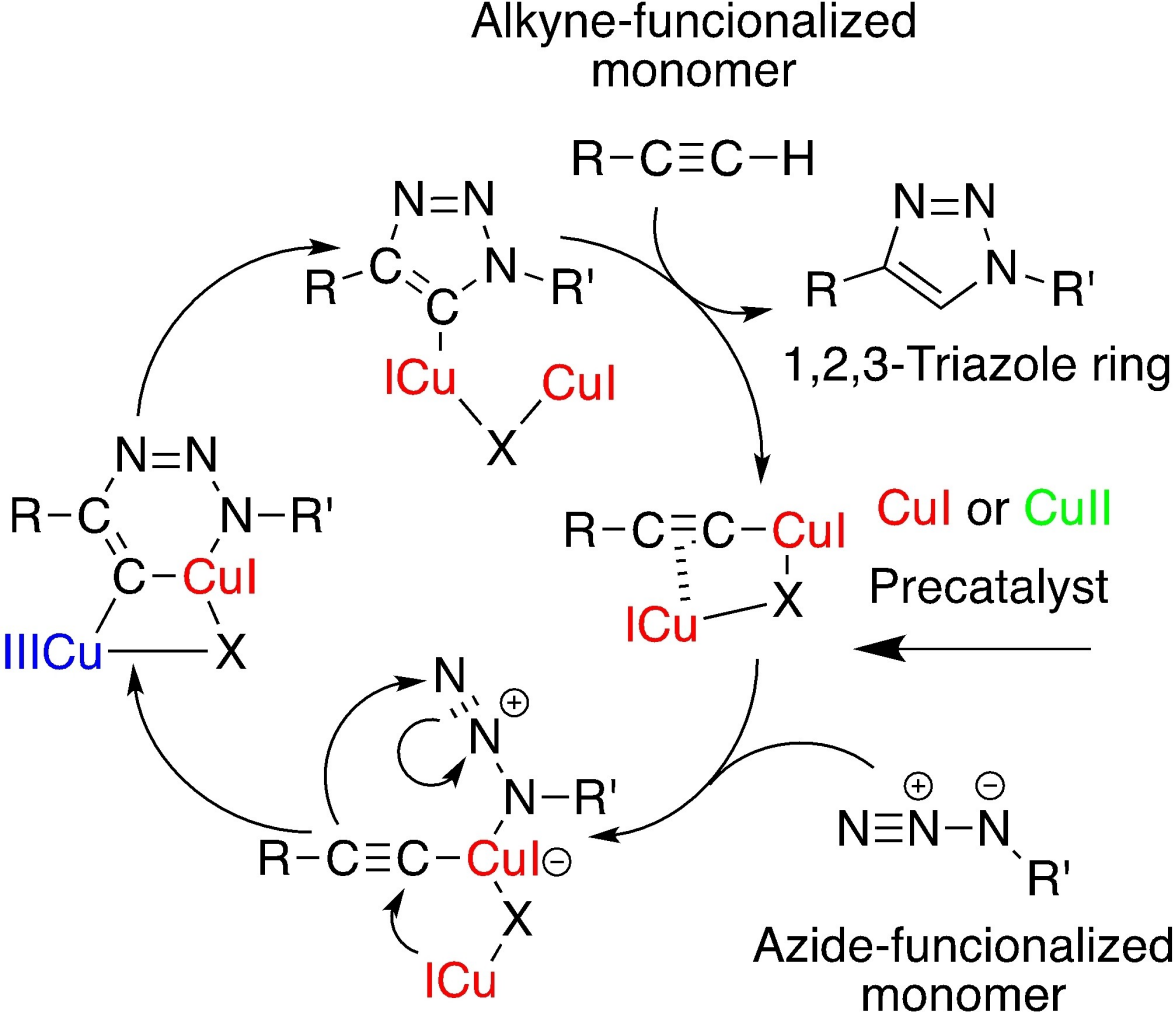
Current proposed mechanism for the CuAAC reaction.

Among the various click reactions, azide‐alkyne cycloaddition (AAC) is one of the most commonly used for cross‐linking purposes and hydrogels synthesis. The reaction couples two entities functionalized respectively by azido and ethynyl groups, regiospecifically affording a 1,2,3‐triazole ring. The catalyst most often used is copper, even though its mechanism of action is not completely clear. Computational analysis suggests that two atoms of copper are introduced into a metallacycle intermediate, followed by ring contraction and formation of cuprous triazolide, which then deprotonates an alkyne to complete the catalytic cycle (Scheme 11).^[108]^

**Scheme 12:**
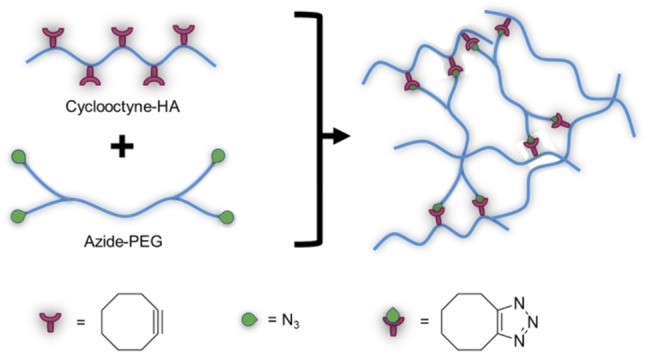
General scheme for cross‐linking via SPAAC.

Yields of the reaction are excellent, and analysis of the residual azide and alkyne species showed that a maximum of 0.2% of unreacted functional groups are present after gel formation.^[109]^ However, Cu catalysed AAC exhibits some limitations, mainly the oxidation of copper that leads to lower efficiency and accumulation of excess catalyst in the hydrogel.^[110]^ Copper ions in HA grafted with different azide cross‐linkers proved to be cytotoxic on murine fibroblast.^[111]^ A possible solution is to use a chelating agent, even though the reduced solubility after chelation of Cu ions also showed cytotoxicity.^[110]^ A more attractive alternative is strain‐promoted azide‐alkyne cycloaddition (SPAAC), employing the inherent ring strain of cyclooctyne to accelerate the reaction without the need of copper catalyst (Scheme 12).^[93]^

For example, polymers such as HYA and PEG functionalized with multi‐cyclooctyne and multi‐azide groups were combined to obtain hydrogels without using copper catalyst. MTT assays on murine or monkey fibroblast showed that both the macromers and the injected cross‐linked hydrogels have excellent cytocompatibility up to a concentration of 5 mg/mL, and in some cases even enhance the cell proliferation.[^112^, ^113^] Further *in vivo* studies after *in situ* formation of the injectable hydrogel in rats displayed high biocompatibility and almost complete disappearance of the hydrogel within 3 days. The potential of the injected hydrogel as an embolic agent was positively evaluated *in vivo* in rabbit ears, being able to act as a thrombus for blocking blood vessels temporarily during local operation.^[113]^ Moreover, SPAAC‐crosslinked hydrogels showed higher compression modulus than most injectable hydrogels, and good swelling and degradation rates.^[112]^ Still, SPAAC‐based cross‐linking, and AAC more in general, is limited by the numerous reaction steps needed for the synthesis of the macromer precursors, which can involve high temperatures and cytotoxic chemicals such as the aforementioned EDC/NHS.[^110^, ^111^] Despite the mild conditions and easy product recovery of the click reaction itself, the strategy is limited by early steps of precursor synthesis.

AAC cross‐linking can be used to synthesise responsive hydrogels that exhibit reversible swelling/deswelling transition in response to different stimuli, such as temperature. Poly(*N*‐isopropylacrylamide) (PNIPAAm) is one of the most investigated thermo‐responsive polymer, because its lower critical solution temperature (LCST) of 32 °C is close to human body temperature, but other polymers such as triblock copolymers based on PEG and other glycols are currently employed.[^114^, ^115^] Example of thermo‐responsive hydrogels was obtained with the Cu‐catalysed click reaction between alkyne modified poly(*N*‐isopropylacrylamide‐*co*‐hydroxyethyl methacrylate) P(NIPAAm‐co‐HEMA) and azide modified cellulose, or between alkyne modified PEG‐PPG‐PEG triblock copolymers and azide modified xylane. The thermoresponsive gels showed a considerable drop in water content when heated above LSCT, while cooling below LSCT causes a rise in swelling ratio. This swelling/deswelling process is completely reversible.[^116^, ^117^]

### 3.3 Dynamic cross‐linking chemistry

**Scheme 13:**
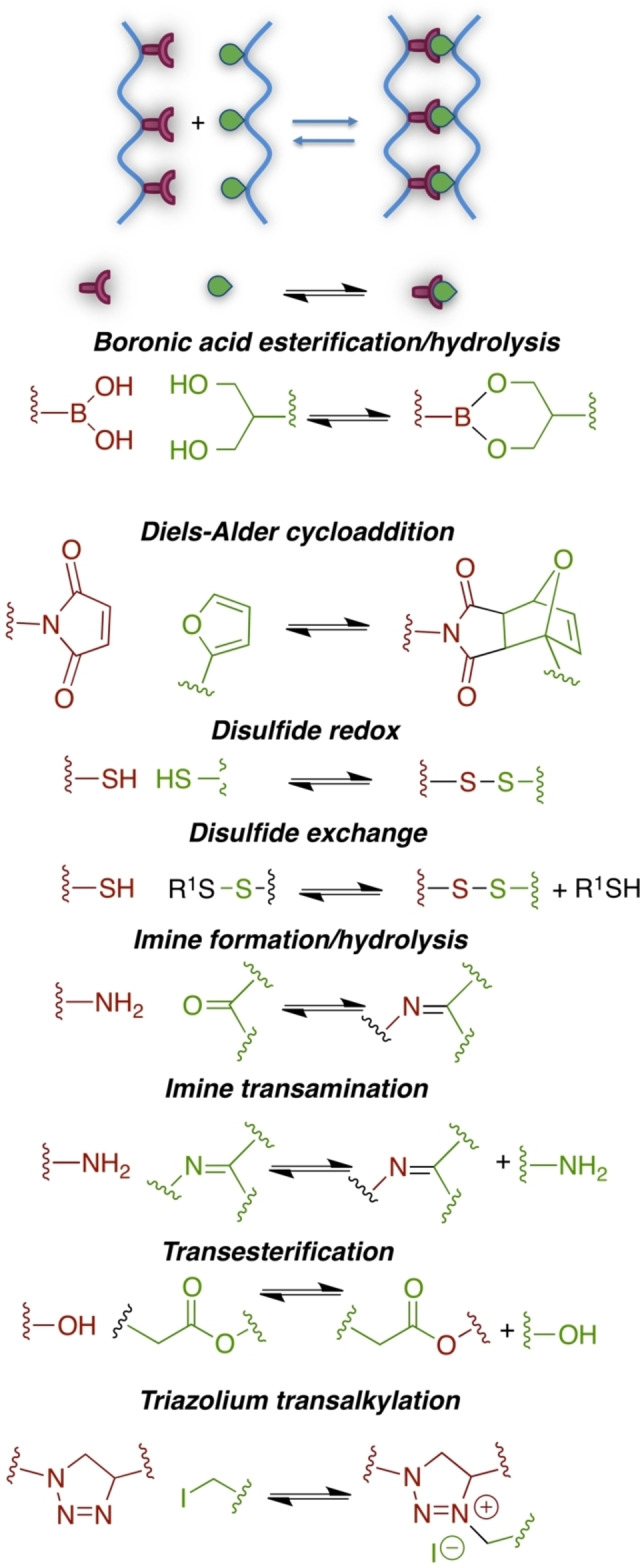
Selected chemical reactions useful for dynamic cross‐linking.

In order to better mimic mechanical features of native ECM and better promote the natural regenerative process, dynamic cross‐links have been proposed. Dynamic covalent cross‐links are innovative and powerful tools,^[118]^ allowing the rearrangement of the hydrogel mechanics and structure as the result of a reversible chemical reaction promoted by a given physical and/or (bio)chemical external stimuli. Different covalent bonds may have a dynamic behaviour, including transesterification, imines hydrolysis/transamination, Diels‐Alder cycloaddition, boronic esters hydrolysis, ligand‐receptor (host‐guest) interactions, disulfide and olefin metathesis, triazolium ion transalkylation, vinylogous urethane transamination (Scheme 13).

Dynamic chemical cross‐linking can be exploited for chemical‐responsive hydrogels. Few examples are reported in the next section.

**Scheme 14:**
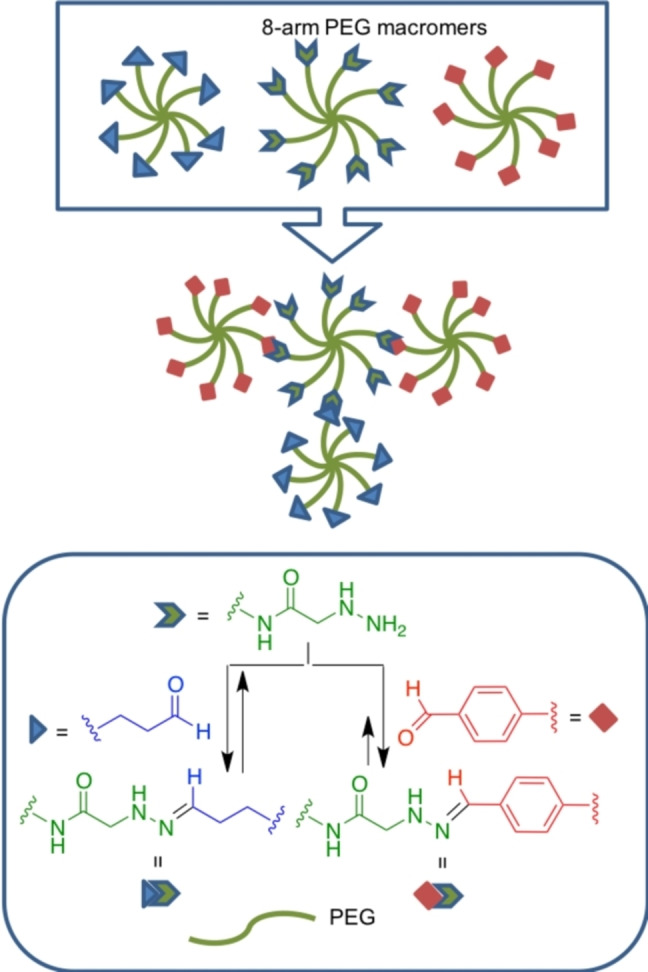
Dynamic cross linking through hydrazine/hydrazone equilibrium.

Richardson and co‐workers exploited the imine transamination equilibrium of hydrazone chemistry for the preparation of dynamic PEG‐based hydrogel scaffolds for cartilage tissue regeneration (Scheme 14).^[95]^ Viscoelastic behaviour based on the equilibrium and Flory‐Stockmayer theory allowed researchers to modulate the average stress relaxation times from hours to months as a function of alkyl hydrazone:benzyl hydrazone ratio, affecting crosslink density. Extracellular matrix deposition by embedded chondrocytes was a result of the hydrazone covalent adaptability and an optimal hydrogel formulation was obtained, where chondrocytes deposited more collagen and more sulfated glycosaminoglycans.

## 4 Stimuli‐responsive hydrogels

**Figure 11:**
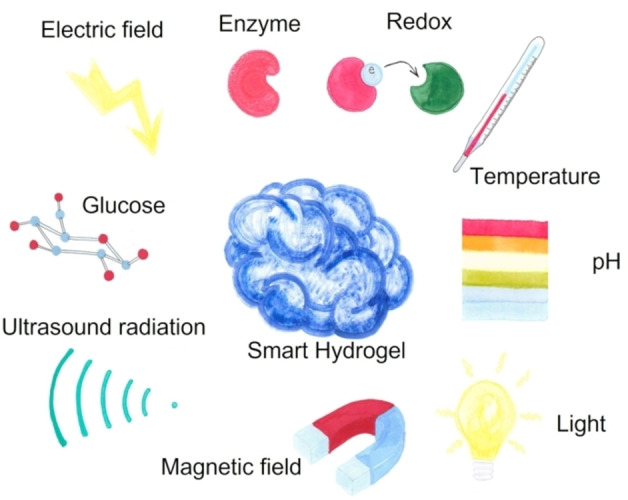
Schematic representation of external stimuli exploited in smart hydrogels.

Besides stable hydrogels offering the advantages of being obtained with robust and straightforward chemistry, and low cost macromers, since the last decade a class of smart/stimuli responsive hydrogels has been proposed. Briefly, smart or stimuli responsive hydrogels may be defined as scaffolds that undergo reversible sol‐gel phase transitions and/or modify their physico‐chemical features in response to changes in their environment. Complex strategies come into play, combining different materials, cross‐linking chemistry, mechanical, topographical and biological cues, together with adaptive cues to the environment. In this frame, smart hydrogels are gaining great interest among researchers and several excellent reviews have been published in recent years.[^8^, ^119^, ^120^, ^121^, ^122^] Roughly 25% of Pubmed hits upon the query “hydrogels” concerns “stimuli responsive hydrogels” in 2021. Smart hydrogels may be responsive to physical (*i. e*., temperature, ultrasounds, electromagnetic fields, light, pressure, mechanical strain), and (bio)chemical stimuli (*i. e*., pH, glucose concentration, ionic strength, ligand‐receptor interactions, enzymes), as schematically represented in Figure 11.

A selection of stimuli responsive hydrogels are cited in the following paragraphs, with main focus on (bio)chemical responsive hydrogels.

### 4.1 Biomolecule responsive hydrogels

Biomolecule‐responsive hydrogels are promising for pre‐clinical and clinical applications, including biosensors and drug delivery platforms. Protein, nucleic acids, carbohydrates are all target biomolecules, however the field is still in its infancy. Several issues hamper a straightforward development of biomolecule‐responsive hydrogels, such as biocompatibility and toxicity. Within this framework, glucose‐responsive devices are of outstanding interest in the biomedical field, given glucose‘s well known involvement in hyperglycemia‐related pathological conditions. Glucose‐sensitive hydrogels are considered as promising on‐demand insulin releasing tools. Three main strategies are commonly used for glucose‐responsive hydrogels.^[123]^

- Phenyl boronic chemistry: 1,2 and 1,3‐diols present in glucopyranose can form reversible boronic esters that can influence hydrogel‐crosslinking and hence its behaviour.^[124]^
- Glucose oxidation: catalase or glucose oxidase eventually entrapped into the hydrogel may mediate glucose oxidation to the corresponding gluconic acid; the acid causes a drop in pH that can modify pH‐responsive cross‐links.^[125]^
- Ligand‐receptor interactions: concanavalin A/glucose recognition^[126]^ may be exploited to trigger hydrogel response to glucose concentration.

**Scheme 15:**
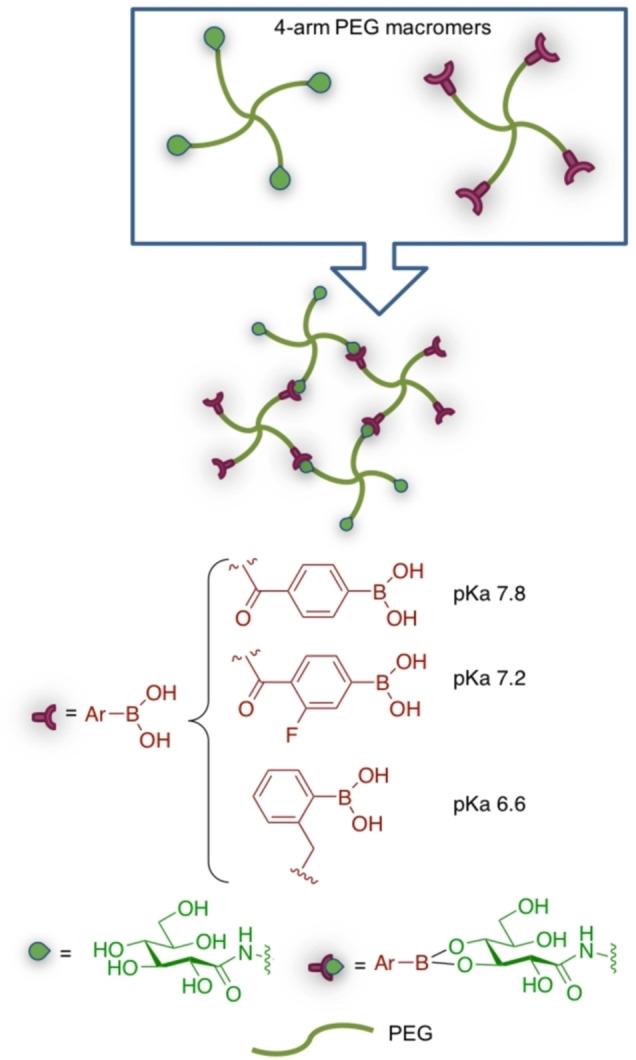
Glucose and pH‐responsive hydrogels based on boronic esters chemistry.

Beside drug delivery applications, glucose‐boronic ester chemistry was elegantly explored by Marozas and coworkers, in order to study cell temporal mechanotransduction by tuning viscoelastic behaviour through controlled chemical cross‐linking of PEG diacrylates, 3‐(acrylamido)phenylboronic acid and methyl 6‐O‐acryl glucoside macromers by 2,3‐glucosyl boronates.^[127]^ The pH‐dependent chemical stability of different boronate esters was exploited by Yesilyurt et al. for the preparation of injectable responsive hydrogels both to glucose and pH, as drug delivery systems and 3D scaffolds for cell culture.^[128]^ PEG macromers suitably derivatized by different aryl boronic acids and by gluconamide (Scheme 15), were synthesised and cross‐linked. Since the different aryl boronic acids differ in their pKa values, cross‐link through boronic esters can occur only at pH above their pKa. Thus, the relationship between pKa of the aryl boronic acid and the environmental pH dictates the extent of cross‐linking. The pH sensitivity of this dynamic covalent bond allows the preparation of hydrogels with pH‐responsive mechanical features. The hydrogel was biocompatible as scaffold for 3T3 fibroblasts cell growth, could be injected subcutaneously with the gelling process occurring after injection and was demonstrated to be glucose sensitive. In fact, in the presence of free glucose increasing concentration, mimicking hyperglycemia in diabetes patients, the boronic acid‐gluconamide cross‐links were broken and substituted by boronic acid‐free glucose esters, thus disrupting the network and allowing the release of drugs (*i. e*. insulin).

### 4.2 Redox‐responsive hydrogels

Oxidative and reductive conditions are frequently found in biological environments, thus they are useful as stimuli for the development of responsive hydrogels. Oxidative conditions for example are related to reactive oxygen species (ROS) involved in pathophysiological states,^[129]^ while reductive conditions may be related to glutathione (GSH) metabolism.^[130]^ Thus hydrogels responding to redox potential are especially interesting for biomedical applications. Particularly sensitive to redox environments and biocompatible functional groups are disulfide bridges that have been studied for this purpose.^[131]^

**Scheme 16:**
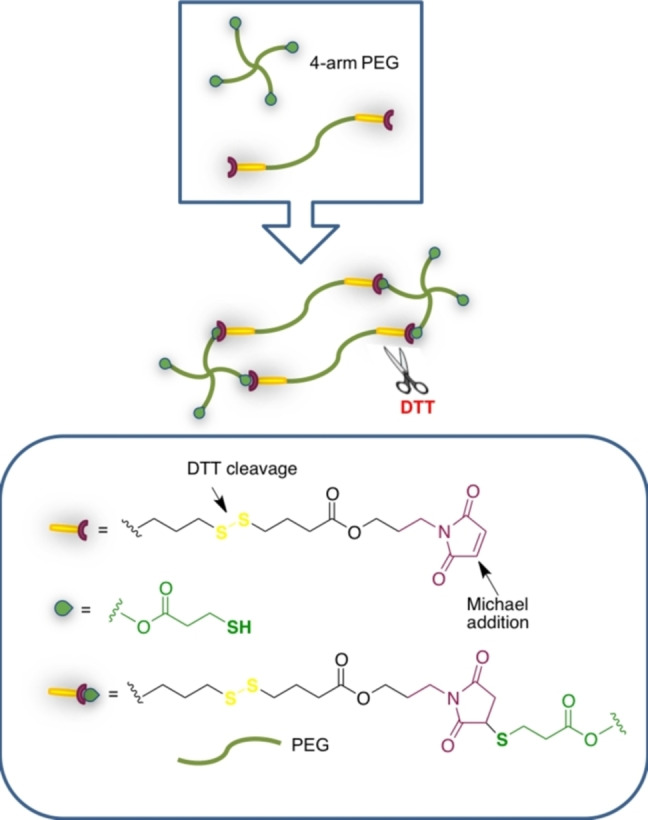
Chemo‐orthogonal chemistry in redox‐responsive hydrogels.

An elegant example exploits click chemo‐orthogonality given by nucleophilic *versus* radical reactivity of thiol groups: nucleophilic behaviour is considered for Michael addition to maleimide groups, while the radical mechanism is involved in disulfide bond reduction.^[132]^ Tetra‐arm thiolated‐PEG was cross‐linked with linear difunctional disulfide‐ and maleimido‐containing PEG (Scheme 16). In very short times, chemoselective reactions between free ‐SH and maleimido groups occurred, without any competing nucleophilic disulfide exchange side reaction. When the hydrogel was placed in reductive conditions (*i. e*., in the presence of a reducing agent such as 4‐dithio‐D,L‐threitol, DTT), the hydrogel degraded. The effectiveness of the responsiveness behaviour was considered for drug delivery. In addition, both the cross‐linked hydrogel and its degradation products resulted cytocompatible when assayed with the L929 fibroblast cell line.

### 4.3 Enzyme‐responsive hydrogels

**Scheme 17:**
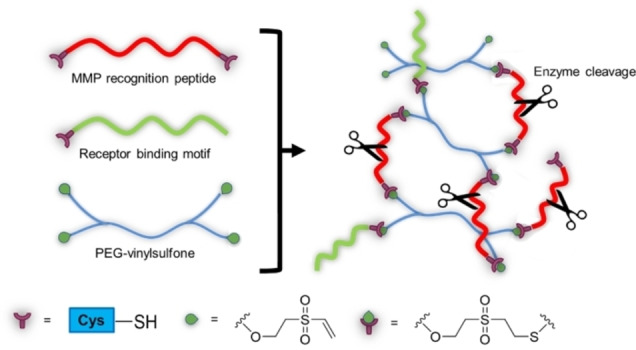
Schematic synthesis of a MMP‐sensitive PEG‐based hydrogel.

Another interesting concept is enzyme‐responsive hydrogels that can undergo network formation, or degradation, altering their swelling and mechanical behaviour. The main application is in drug delivery, as controlled and selective release platforms.[^93^, ^109^] Enzyme‐responsive hydrogels usually include peptide sequences either as part of the polymer backbone or as cross‐linkers, or functional groups that can be recognized and react with cell‐secreted or cell‐activated enzymes. In a pioneer study, a metalloproteinase (MMP)‐sensitive PEG‐based hydrogel was used as scaffold for chondrocyte growth. The click reaction between PEG‐vinylsulfone macromers and thiol‐ended MMP recognition peptide, conducted *in situ*, gave the smart hydrogel. A peptide with a receptor‐binding motif was also linked with the same method, to confer adhesiveness to the gel (Scheme 17).^[133]^ The controlled degradation of the hydrogel matrix by MMPs secreted by the chondrocyte culture allowed the better dispersion of matrix components and migration of cells inside the hydrogel, and also affected the gene expression profile leading to a four fold higher collagen production than the control. Very interestingly, no boundaries between the cell‐derived‐matrix and the hydrogel was observed.^[134]^

A desirable approach for the synthesis of enzymatically degradable hydrogels is *via* click reactions. A bis‐azido peptide with the D‐Ala‐Phe‐Lys moiety, specific for trypsin and plasmin hydrolysis, was combined with a PEG‐alkyne derivative via CuAAC, and the resulting hydrogels was completely dissolved by trypsin within 80 hours.^[135]^ In another example, the protease‐sensitive peptides were functionalized with di‐alkynes and then combined with azide‐terminated PEG via CuAAC. Papain was used as a model enzyme to recognise these peptides and cleave the hydrogel, and time‐course analysis proved that the soluble fraction molecular weight quickly reached that of the PEG macromer.^[136]^

### 4.4 Antigen/antibody‐responsive hydrogels

As previously reported, hydrogels that undergo changes upon external physical and chemical stimuli are promising for biomedical applications, however it would be interesting to have materials that respond to a specific biomacromolecule (*i.e*, proteins and polysaccharides), as they would be able to mimic some natural feedback mechanism.[^137^, ^138^] For this purpose, materials with the ability to swell and unswell in the presence of a specific antigen were synthesised. The antibodies or the antigens themselves can be physically trapped or chemically conjugated to the hydrogel, or included into macromers, then paired to generate reversible cross‐linking in the hydrogel network.^[139]^

**Scheme 18:**
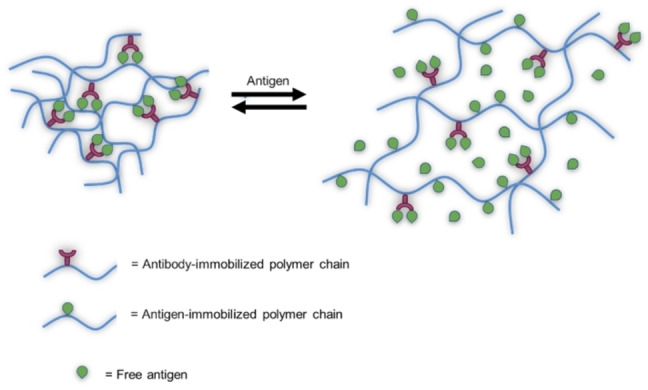
Swelling/deswelling behaviour of smart hydrogel in response to different concentrations of antigen.

For example, dextran macromers containing fluorescein‐5‐isothiocyanate (FITC) as the antigen, and anti‐FITC‐IgG as the antibody were synthesised *via* divinyl sulfone chemistry; antigen/antibody cross‐linking afforded the antigen‐responsive hydrogel.^[139]^ The addition of free antigen (FITC) dysrupt cross‐linking, greatly increasing the swelling ratio, because it competes with the cross‐linking bonds among the polymer chain of the gel. This feature can be exploited for drug delivery in response to a specific antigen (Scheme 18).^[137]^ In addition, the antibody‐responsivness was also assayed. It is supposed that native and free antibodies will display a much stronger binding to the antigens than grafted ones, because of partial denaturation of the protein after the chemical modification needed for grafting to the macromer. This higher affinity of the native antigen was demonstrated by the complete dissociation of the cross‐linking bonds leading to hydrogel disruption and to high permeation of water.

Instead of linking the entire antibody to the macromer chain, an alternative is to use its fragments, to allow better control over size and composition of the hydrogel. Fragment antigen‐binding regions (Fab’) from monoclonal anti‐fluorescein antibody (IgG2a) was co‐polymerised with *N*‐isopropylacrylamide (NIPAAm) and *N,N’*‐methylenebis(acrylamide) (MBAAm) using redox initiators, or was reacted with a maleimido group containing monomer and then co‐polymerised with *N*‐(2‐hydroxypropyl)methacrylamide (HPMA).^[140]^ In these cases, the antigen‐sensitive hydrogels could detect target antigen with high precision and sensitivity. In particular, it was proved that the Fab’ fragments could bind receptors on human ovarian carcinoma cells, leading to pinocytosis of the polymer in the cytoplasm. The fast accumulation resulted in an efficient inhibition of cancer cells growth.^[141]^ The use of free Fab’ fragments would result in fast clearance from the blood,^[142]^ while the copolymer‘s higher hydrodynamic size overcomes this problem, on top of giving higher protection towards proteolysis.

## 5 Mechanical insights

Mechanical properties of hydrogels are important design criteria to be considered for engineering of scaffolds in the field of tissue engineering. Indeed, in this field, both macroscale and microscale mechanical behaviour is a driving force in regulation of cell fate: biomechanical signals affect cellular interactions mediated by the extracellular matrix (ECM) guiding the cell phenotype.^[143]^ As the ECM in a tissue is responsible for holding cells in place while also providing diffusional access for cell‐signalling cues, nutrients and other moieties, these functions are addressed by hydrogels, as scaffold, in a cell culture environment.

From a mechanical standpoint hydrogels, in some cases, can act like a rubber and their properties rely on time‐independent recovery of polymer chains in the network after loading removal. In this case it is possible to describe their behaviour by the theory of time‐independent rubber elasticity where they respond to stresses instantly, with a complete recovery after loading removal. The elastic behaviour is characterised over properties such as Young's modulus (E), tensile strength, failure strain and compressive strength. Elasticity refers to the capability of a material to deform instantaneously when undergoing a mechanical loading. E describes the resistance of an elastic material to stress and is ordinarily identified as the slope of the linear region of the stress–strain curve. Where the stress is defined in terms of force applied per unit area of the hydrogel and the strain as the ratio of the change in length of the hydrogel sample to the original length caused by stress. Tensile strength is defined as the stress at break point during an elongation of hydrogel. Compressive strength refers to the capacity to endure the loading induced to decrease the size. Common techniques used for measuring elastic properties of hydrogels involve tensile testing, compression either confined or unconfined and indentation testing. For the tensile test, the hydrogel sample is placed between two clamps, and one end of the hydrogel is extended by different loads and extension rates. Tensile testing is mostly uniaxial and generates forces along one axis, while 2D axial forces are also possible. These methods have been extensively used to characterise mechanical behaviour of various hydrogels in different shapes such as cylinders, strips or rings. The applied force and elongation of the hydrogel are used to obtain stress‐strain curve and derive the mechanical parameters defined above. Nevertheless, at certain conditions, such as a lower temperature or a protracted exposure to mechanical loading, they can exhibit a viscoelastic behaviour, where the response to stresses is instantaneous and where the applied stress results in an instantaneous followed by a viscous, time‐dependent strain. In the first case mechanical properties of hydrogel are described using the theory of time‐independent elasticity while in the second condition the time‐dependent viscoelasticity theory is used. Based on these theories, equations have been derived to characterise mechanical behaviour of hydrogels in different states and correlate mechanical properties of polymers with their structural networks. Unlike purely elastic time‐independent behaviour, a viscoelastic hydrogel has both an elastic component and a viscous component. The viscosity gives the hydrogel a strain rate dependence on time. If ideal elastic hydrogels do not dissipate energy when a load is applied and eventually removed, a viscoelastic hydrogel dissipates energy when a load is applied, then removed. Hysteresis is observed in the stress–strain curve, with the area of the loop being equal to the energy lost during the loading cycle. Specifically, viscoelasticity is a molecular rearrangement of the polymer network within the hydrogel. When a stress is applied the long polymer network rearranges and when the original stress is removed the hydrogel returns to its original state. The hydrogel creeps, which gives the prefix visco‐, and the hydrogel fully recovers, which gives the suffix ‐elasticity. Rheology is the mechanical characterization technique useful for studying characteristics of viscoelastic hydrogels. The working principle of a rheometer relies on measuring the stress response resulting from inducing a sinusoidal shear deformation (with a certain frequency w) to a hydrogel sample. The sample is usually placed between two parallel plates, between a cone and plate or between coaxial cylinders. At the given frequency the visco‐elasticity is characterised by measuring the loss modulus and the storage modulus, respectively. The hydrogel has an ideal elastic behaviour if the stress is always in phase with the applied sinusoidal strain. The stress remains proportional to the strain, and the proportionality constant is defined as the shear modulus of the hydrogel. In contrast, if the hydrogel behaves like a purely viscous material, the measured stress is proportional to the rate of strain, where the proportionality constant is the viscosity. Viscoelastic hydrogels exhibit both elastic and viscoelastic characteristics when undergoing deformation. Porosity is another important feature of hydrogels that contributes to their mechanical properties. In particular, free volume percentage, pore size, interconnectivity and surface properties of pores are the most meaningful parameters. In general the higher porosity and pore size the lower the mechanical properties of hydrogel scaffold. Moreover, the delivery of nutrients and oxygen to the cells encapsulated within a hydrogel are positively affected in case of a vast amount of void volume, that is porosity. Consequently, a competition between mechanical performance and biological performance exists that could be alleviated by hydrogels with an intrinsically high mechanical strength. The other important property of hydrogels is their degradation which influences the biomechanics of hydrogels, and the behaviour of resident cells. The degradation of hydrogels may also cause weakening of hydrogels which implies that the rate of hydrogel degradation should be equal to cellular formation rate of new ECM.

**Figure 12:**
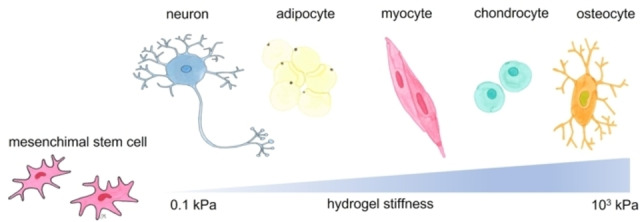
Hydrogel stiffness may influence stem cell differentiation to diverse cell lines.

The stiffness (the elastic modulus) is one of the most widely studied mechanical properties in regenerative medicine applications.^[144]^ In this regard, the most widely used materials for tuning stiffness are polyacrylamide, polyethylene glycol and alginate‐based hydrogels where by modulating the degree of crosslinking or the molecular weight and macromer content it is possible to vary their stiffness from several Pa to several tens of kPa. The first noteworthy example regards the differentiation of stem cells towards a specific lineage driven by the matrix stiffness (Figure 12).

The study by Engler and co‐workers, dates back to 2006, constitutes the phenomenological evidence of cell preference regarding external stimuli for differentiation towards a specific lineage.^[145]^ In this study human mesenchymal stem cells spontaneously differentiated into neurons on soft polyacrylamide hydrogels (with an elastic modulus of 0.1–1 kPa), while they differentiated into muscular and bone‐like lineages on moderate and hard gels (with an elastic modulus of 8–17 kPa and 25–40 kPa, respectively). The preference of neurons for compliant substrates has also been observed in neurite branching and springing. Comparing the shape of neurons cultured on compliant and rigid substrates, they exhibited more branched and elongated sprouts on the compliant substrate. Analogously, by modulating the stiffness of alginate hydrogels, mesenchymal stem cells differentiate into osteoblasts in case of high stiffness, while on hydrogels with low matrix stiffness, stem cells tend to differentiate into chondrocytes or adipocytes.[^144^, ^146^] As well as studies on the effect of the hydrogel stiffness on cell differentiation, the study of hydrogels with different stiffness in tissue repair has become a research hotspot. Stiffness‐tunable hydrogels can be obtained by changing the crosslinking degree of gels, varying external conditions, changing the molecular weight of materials, or adding nanomaterials. The concentration of crosslinking agent on polyacrylamide hydrogels has been investigated for renal tissue regeneration showing that varying the concentration from 0.5 μL to 30 μL the stiffness becomes 719 Pa and 5.9 kPa, respectively.^[147]^ Regarding the effect of external stimuli these could be: pH, magnetic field, UV irradiation, or temperature.^[146]^ As an example, regarding the latter stimulus, multicomponent hydrogel for wound healing hydrogels composed by block copolymer F127 and alginate has been evaluated. The elastic modulus is 2.9 kPa (at 15 °C) and it becomes 40 and 50 kPa at 25 °C and 40 °C, respectively.^[148]^ Regarding the effect of the molecular weight on the hydrogel *N*‐(2‐aminoethyl)maleimide trifluoroacetate salt/hyaluronic acid with different molecular weight hydrogels have been investigated for the regeneration of cartilage tissue by culturing bone marrow‐derived mesenchymal stem cells.^[149]^ Three hyaluronic acid species with different molecular weights–low (4 kDa), medium (10 kDa), and high (90 kDa) have been selected to regulate the physical hydrogel properties. Here, the smaller the molecular weight, the lower the stiffness of the hydrogel (0.2 kPa for the 4 kDa hydrogel and 1 kPa for the 90 kDa hydrogel). Finally considering the inclusion of particulates in the hydrogel matrix, a hybrid hydrogel made by methacrylated gelatin, chitosan and polyhedral oligomeric silsesquioxane has been proposed for *in situ* bone regeneration.^[150]^ This hydrogel shows advantageous characteristics as it exhibits enhanced stiffness by the dispersed inorganic units (108 kPa), the bio‐macromers (chitosan and gelatin) infer an appropriate biodegradation behaviour, and an accelerated *in situ* bone regeneration is observed, too.

The mechanical behaviour of hydrogels has long been recognised as a fundamentally important driving force not only for cell differentiation but also for tissue repair and inflammatory response. In this regard the change of hydrogel stiffness is mediated by some mechanically sensitive proteins Rho and YAP, which cause the secretion of reactive oxygen species (ROS). ROS is a general term for a class of oxygen‐containing active chemicals in the body and the increased expression of ROS can increase the content of mesenchymal stem cells related to the protein secretome and control the related physiological processes in the tissue repair [A].^[151]^ In an experiment of stiffness‐ induced stem cell behaviour, it was shown that ROS can act as an upstream signal to regulate cell behaviour and secretome: ROS expression by mesenchymal stem cells decreased with the increase of hydrogel stiffness.^[152]^ Moreover, by increasing the hydrogel stiffness it is possible to change the phenotype of macrophages, transforming them from anti‐inflammatory type to pro‐inflammatory one, and thus promoting tissue repair by regulating related inflammatory reactions and immune processes.^[153]^

## 6 Conclusions

Since the introduction of hydrogels, their design criteria were heavily modified in order to best suit different applications and their needs. In particular, in the biomedical field the chemical, physical and biological inertness, given by chemical and metabolic stability of macromers and cross‐linking chemistry were initially relevant issues. Over the years the increased knowledge in the biological processes underlying tissue engineering, pathological states, and drug uptakes, flanked with the progress in advanced chemical strategies such as click‐chemistry, and dynamic chemical bonds allowed the production of smart/reversible hydrogels. Inertness is now substituted by chemical, physical and metabolic responsiveness. The field is fastly growing and research is still ongoing. Main limitations to market applications of these innovative platforms are biocompatibility, and high costs due to multistep chemistry and macromer production. However the versatility of these platforms open a huge space of combinations that could match applications and market development.

## Conflict of interest

The authors declare no conflict of interest.

## Biographical Information

*Federico Acciaretti graduated in Industrial Biotechnology at the University of Milano ‐Bicocca, Italy, in 2021 with a work on the synthesis of sugar‐based building‐blocks from dairy industry wastes for polyesters synthesis. He received a research scholarship in the group of Prof. Laura Cipolla on the same subject. He participated and won the call for paper 2019 “Hands on Vinyl: Students of Today, Experts of Tomorrow”. His current research interest focuses on biopolymers, and the upcycling of wastes into added‐value chemicals, by chemical or biotechnological exploitation*.

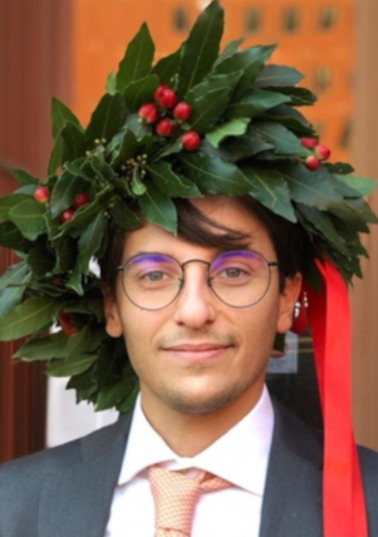

## Biographical Information

*Simone Vesentini since 2015 is Associate Professor in Industrial Bioengineering at the Department of Electronics, Information and Bioengineering at the Politecnico di Milano. His research interests include soft tissue modeling and biomolecular modeling on the structure‐function relationship in extracellular matrix in physiological and pathological conditions, design of bioinspired materials, use of by‐product of natural origin for smart materials. He is author of about 50 scientific publications in the field of nanobiomechanic and molecular modeling*.

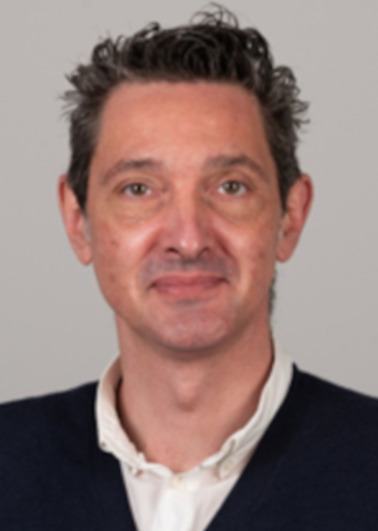

## Biographical Information

*Laura Cipolla is currently Associate professor in organic chemistry at the Department of Biotechnology and Biosciences, University of Milano – Bicocca. She received her PhD in chemistry (1996) from the University of Milano, working on carbohydrates and their mimics synthesis. Her current research interests encompass biomaterials for regenerative medicine, and material design from renewable resources for different applications. She authored about 150 publications in the field of carbohydrate chemistry, and materials for biomedical applications*.